# Nonlatching positive feedback enables robust bimodality by decoupling expression noise from the mean

**DOI:** 10.1371/journal.pbio.2000841

**Published:** 2017-10-18

**Authors:** Brandon S. Razooky, Youfang Cao, Maike M. K. Hansen, Alan S. Perelson, Michael L. Simpson, Leor S. Weinberger

**Affiliations:** 1 Laboratory of Virology and Infectious Disease, The Rockefeller University, New York, New York, United States of America; 2 The Gladstone Institutes (Virology and Immunology), San Francisco, California, United States of America; 3 Biophysics Graduate Group, University of California, San Francisco, San Francisco, California, United Sates of America; 4 Center for Nanophase Materials Sciences, Oak Ridge National Laboratory, Oak Ridge, Tennessee, United States of America; 5 Bredesen Center for Interdisciplinary Research and Graduate Education, University of Tennessee, Knoxville, Tennessee, United States of America; 6 Theoretical Biology and Biophysics (T-6), Los Alamos National Laboratory, Los Alamos, New Mexico, United States of America; 7 Center for Nonlinear Studies (CNLS), Los Alamos National Laboratory, Los Alamos, New Mexico, United States of America; 8 Department of Biochemistry and Biophysics, University of California, San Francisco, San Francisco, California, United States of America; 9 QB3: California Institute of Quantitative Biosciences, University of California, San Francisco, San Francisco, California, United States of America; 10 Department of Pharmaceutical Chemistry University of California, San Francisco, San Francisco, California, United States of America; The Hebrew University of Jerusalem, Israel

## Abstract

Fundamental to biological decision-making is the ability to generate bimodal expression patterns where 2 alternate expression states simultaneously exist. Here, we use a combination of single-cell analysis and mathematical modeling to examine the sources of bimodality in the transcriptional program controlling HIV’s fate decision between active replication and viral latency. We find that the HIV transactivator of transcription (Tat) protein manipulates the intrinsic toggling of HIV’s promoter, the long terminal repeat (LTR), to generate bimodal ON-OFF expression and that transcriptional positive feedback from Tat shifts and expands the regime of LTR bimodality. This result holds for both minimal synthetic viral circuits and full-length virus. Strikingly, computational analysis indicates that the Tat circuit’s noncooperative “nonlatching” feedback architecture is optimized to slow the promoter’s toggling and generate bimodality by stochastic extinction of Tat. In contrast to the standard Poisson model, theory and experiment show that nonlatching positive feedback substantially dampens the inverse noise-mean relationship to maintain stochastic bimodality despite increasing mean expression levels. Given the rapid evolution of HIV, the presence of a circuit optimized to robustly generate bimodal expression appears consistent with the hypothesis that HIV’s decision between active replication and latency provides a viral fitness advantage. More broadly, the results suggest that positive-feedback circuits may have evolved not only for signal amplification but also for robustly generating bimodality by decoupling expression fluctuations (noise) from mean expression levels.

## Author summary

A central and recurring feature of cell-fate regulating circuits is their ability to generate bimodal expression—2 alternate expression states that exist simultaneously—with each state corresponding to a different cell fate. To understand the mechanisms enabling bimodality in a natural decision-making circuit, we examined HIV’s fate-selection circuit, the Tat circuit. This bimodal circuit is sufficient and necessary to generate a bet-hedging decision between 2 alternate HIV fates: active viral replication and long-lived dormancy (proviral latency). The dormant state, which is resistant to most antiviral drugs, is the primary clinical barrier to curing an HIV infection. While the canonical role of positive feedback is to amplify a signal, surprisingly, we find that the HIV transactivator of transcription (Tat) positive-feedback architecture is instead optimized to expand the regime of HIV expression bimodality. From an evolutionary perspective, the results suggest that positive-feedback circuits may have evolved to robustly generate bimodality in certain contexts, and, given the rapid evolution of HIV, the presence of a circuit optimized to robustly generate bimodal expression patterns appears to support the hypothesis that HIV’s active-versus-latent decision confers viral fitness.

## Introduction

**Fig 1:**
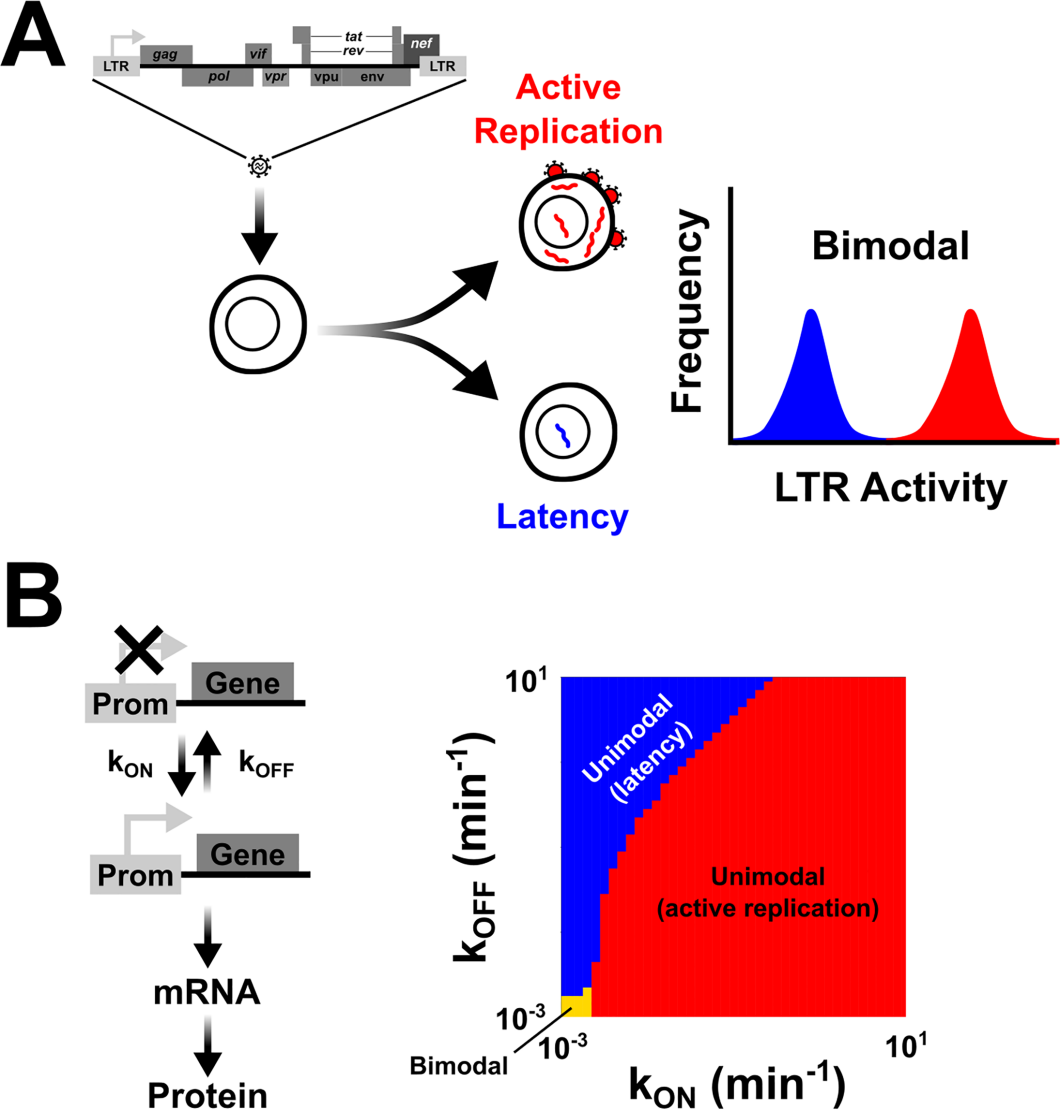
The mechanistic problem underlying bimodal fate-selection programs: Promoter toggling is theoretically sufficient to generate bimodality, but only in a narrow parameter regime. (A) A simplified fate-selection decision in HIV. Upon infection of a CD4^+^ T lymphocyte, HIV either enters into an active state of replication (red), producing viral progeny and destroying the host cell, or enters into a quiescent state of silenced gene expression termed proviral latency (blue). This fate bifurcation between active replication and latency is not controlled by the cell state [27] but rather by an HIV gene-regulatory program that can generate bimodal gene-expression distributions from its long terminal repeat (LTR) promoter. (B) The LTR is accurately described by a 2-state promoter model (e.g., random telegraph models) in which the LTR switches between an inactive (represented by Prom-Gene that is crossed out, top) to an active (represented by Prom-Gene) state of expression at rate k_ON_. In the active state, mRNAs are produced, before the promoter flips back to the inactive state at rate k_OFF_. Promoter toggling between these active and inactive states can produce bimodal distributions in gene-expression products, but only within a restricted regime of phase space. Each parameter set was checked to see if it generated unimodal latency (blue), unimodal active replication (red), or bimodality (orange), as described in the Materials and methods section. For the modality analysis, each mode was required to contain at least 0.1% of the population; otherwise, the parameter set was determined to produce a unimodal population.

Bimodality is a recurring feature in many biological fate-selection programs [1], such as the HIV active-versus-latent decision (Fig 1A). Bimodal expression is a population-wide distribution pattern comprises 2 gene-expression modes, each corresponding to a specific fate path [2]. The mechanisms that can generate bimodal phenotypes have long been studied, and the architecture of underlying gene-regulatory circuits appears to be a key driver of bimodality [3–11]. Classically, bimodality has been associated with deterministic bistability in gene circuits [12–15]. Deterministic bistability requires ultrasensitive input-output relations and can result from nonlinear positive feedback (i.e., Hill coefficient > 1) on a constitutively expressed promoter [16,17]. However, many promoters are nonconstitutive and instead toggle between inactive and active expression states, generating episodic bursts of mRNA production (for review, see [18]). The finding that promoters undergo episodic bursts of expression led to a proposal that this toggling alone could generate bimodality without deterministic bistability. Unlike constitutive expression, toggling increases the degrees of freedom in a system [19], and if promoter toggling occurs relatively slowly, the resulting expression bursts can potentially produce bimodality independent of ultrasensitivity [19,20]. However, the promoter toggling kinetics required to generate bimodality appeared to be in a small portion of the experimentally observed regime [18,21–23], with experimental measures of intrinsic promoter toggling exhibiting kinetics that are typically too fast to produce bimodal expression patterns (Fig 1B)—specifically, the measured promoter toggling rates were greater than the per capita protein and mRNA decay rates [18,24,25]. Nevertheless, synthetic positive-feedback circuits that slowed toggling could induce bimodality [26]. Thus, while computational models showed that promoter ON-OFF toggling was sufficient for bimodal expression [20] and synthetic transcriptional circuits lacking bistable feedback could generate bimodal expression [26], it remained unclear how natural biological circuits exploit this mechanism to generate bimodality without bistability. Here, we determine if promoter toggling can intrinsically generate bimodal distributions in a natural biological system (i.e., HIV) and the potential physiological relevance.

We focus on HIV as a physiological model system for expression bimodality driving a decision-making process (Fig 1A). Upon infection of a CD4^+^ T lymphocyte, HIV undergoes a fate-selection decision, either actively replicating to produce viral progeny and destroy the host cell or entering a long-lived quiescent state called proviral latency [28,29]. A viral gene-regulatory circuit is both necessary and sufficient to drive HIV fate selection [10]. At the core of this decision-making circuit is a virally encoded transcriptional positive-feedback loop comprises a single HIV protein—the transactivator of transcription (Tat)—that amplifies expression from the virus’s only promoter, the long terminal repeat (LTR) promoter. Molecularly, this positive-feedback loop functions because the LTR is a relatively weak promoter, in the absence of Tat, with RNA polymerase II (RNAPII) elongation stalling approximately 69 nucleotides after initiation [30]. Tat transactivates the LTR by binding to a short, approximately 69-nucleotide-long RNA-hairpin loop called the Tat-activation RNA (TAR) loop and recruiting the positive transcriptional elongation factor b (pTEFb)—principally composed of CDK9 and cyclinT1—which hyperphosphorylates the carboxy-terminal domain (CTD) of RNAPII, thereby relieving the RNAPII elongation block [30,31]. Thus, Tat acts much like a bacterial antiterminator enhancing transcriptional elongation rather than initiation.

Importantly, minimal LTR-Tat positive-feedback circuits are sufficient to generate bimodal expression patterns [32], and in the full-length viral context, this circuit is both necessary and sufficient to drive HIV’s active-versus-latent decision [27]. There are 2 specific quantitative features of the Tat-LTR feedback circuit that are curious, given its obligate role in viral fate selection. First, unlike many other positive-feedback circuits that control phenotypic decisions [33,34], the Tat positive-feedback loop is noncooperative (Hill coefficient ≈ 1) and not deterministically bistable [35]. Second, the LTR promoter itself displays large episodic expression bursts toggling between ON and OFF states at virtually all integration sites throughout the human genome [24,36,37], raising the possibility that the LTR itself may be sufficient to generate bimodal expression patterns independent of Tat feedback.

In this study, we construct minimal circuits to examine if the LTR itself is capable of generating bimodal expression patterns in the absence of Tat feedback and then computationally examine the precise role of Tat positive feedback in bimodality. The results indicate that the LTR is intrinsically capable of generating bimodal ON-OFF expression even in the absence of feedback but that Tat feedback shifts and expands the regime of LTR bimodality into physiological ranges by slowing LTR toggling. In fact, the architecture and parameters of the Tat circuit appear optimized to robustly generate bimodal expression. Given the rapid evolution of HIV, the presence of a circuitry that appears optimized to slow promoter toggling and generate bimodality may be consistent with the hypothesis that the circuit has been selectively maintained and that bimodal expression (between active replication and latency) provides a viral fitness advantage [38].

## Results

### LTR promoter toggling is capable of generating bimodality in the absence of feedback

Previous studies demonstrated that Tat positive feedback can generate bimodal expression patterns from the HIV LTR [32]. However, given the large, episodic bursts of expression that characterize LTR activity [24,36,37], we set out to test if the LTR was capable of bimodal expression, even in the absence of feedback (i.e., whether feedback was dispensable for bimodality, possibly having an orthogonal function in HIV). Analysis of experimental and computational literature reports indicated that the regime for generating bimodality through promoter toggling alone fell outside the experimentally observed values of LTR toggling but that slightly slower LTR toggling transitions might generate bimodality without feedback (Fig 1 and S1 Fig).

**Fig 2:**
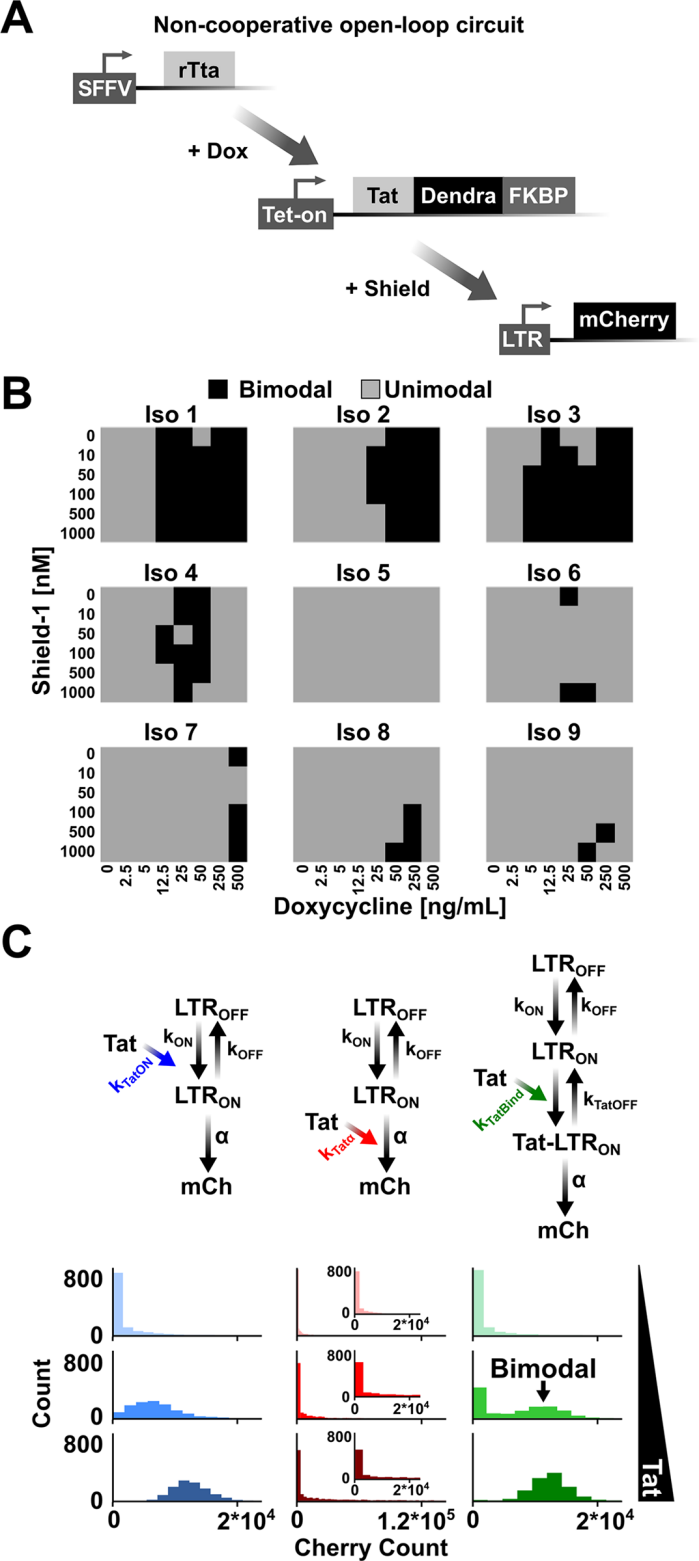
Long terminal repeat (LTR) promoter toggling is sufficient to generate bimodality and control HIV fate. (A) Schematic of the open-loop HIV circuit. Doxycycline addition induces transcription from the Tet-ON promoter. Shield-1 addition controls the stability of the transactivator of transcription (Tat) fused to Dendra-FKBP fusion protein. Tat induces transcription from the HIV LTR. (B) The (Iso) term represents an independent isoclonal population; consequently, each cell within a clone has the same integration site for the LTR. Nine Iso populations were exposed to 48 different doxycycline and Shield-1 conditions (S2 and S3 Figs and S1–S23 Data), and bimodality was tested for by the Hartigan Dip Test [39] (the threshold for determining bimodality was *p* < 0.3, agreeing with an independent test, S3 Fig and S24 Data). Gray squares indicate populations that were determined to be unimodal, and black squares represent bimodal populations. (C) Open-loop stochastic model of Tat transactivation of the LTR by 1 of 3 mechanisms. Left column, increasing burst frequency by promoting transitions into the LTR_ON_ state (left, increasing k_ON_, blue); middle column, increasing burst size by increasing transcriptional efficiency (middle, increasing α, red); and right column, increasing burst size through addition of a third promoter state (effectively inhibiting k_OFF_, green arrow). Note that for the model in which Tat effectively modulates k_OFF_ (right), there is an additional production of mCh from the LTR_ON_ state (arrow not shown) at rate α so that changes in burst sizes can be generated without altering transcriptional efficiency. Model equations and details are presented in S1–S3 Tables. Plotted histograms are steady-state results of 1,000 simulations (at 1,000 hours) showing that slowing promoter toggling by inhibiting transitions into the active state is sufficient to generate bimodal distributions (i.e., right column, middle panel). Insets: Zoom of α modulation so the scale of the *x*-axis matches the k_ON_ (left column) and k_OFF_ (right column) modulation graphs (S1 Data).

To test this prediction that Tat feedback was dispensable for bimodality, HIV circuitry was refactored to split the Tat positive-feedback loop [27] into open-loop parts (Fig 2A). This minimal circuit system allows Tat concentrations to be modulated by doxycycline (Dox) and Tat protein stability to be tuned through Shield-1 addition [27]. As Tat is fused to Dendra, the Tat concentrations can be quantified, while LTR activity is simultaneously tracked in single cells. This open-loop doxycyline-inducible circuit was integrated into T cells by viral transduction, and cells were exposed to varying concentrations of activator (Dox) and Tat proteolysis inhibitor (Shield-1)—generating approximately 48 unique unimodal Tat inputs to the LTR (S2 and S3 Figs and S1–S23 Data). Expression profiles from the LTR are all unimodal in the absence of Tat (S2 Fig), in agreement with previous findings [32,36,37]. However, in striking contrast, the presence of Tat induces bimodality from the LTR despite the lack of cooperativity or feedback in this open-loop system (Fig 2B, S2 and S3 Figs and S1–S23 Data). In other words, despite a fixed, unimodal concentration of active Tat transactivator, bimodal LTR distributions can be generated, and single-cell time-lapse microscopy confirms that the activity of the LTR is dependent on Tat input (S4 Fig and S24 Data). From the known requirements for bimodality to arise from a toggling promoter (Fig 1), the data suggest that LTR toggling becomes sufficiently slow in the presence of Tat to produce bimodal expression patterns, even in the absence of positive feedback.

### Independent of feedback or cooperativity, LTR promoter toggling is sufficient to control full-length HIV fate

The bimodality in the minimal open-loop system (Fig 2) represents the 2 fate paths of the virus—active replication and proviral latency [40]—and suggests that positive feedback may also be dispensable for controlling viral fate in full-length HIV. Importantly, results from a Tat-deficient full-length HIV virus [27], where Tat is introduced in trans (S5 Fig), confirm that Tat feedback is not required to select between alternate HIV fate paths. Thus, unlike other decision-making circuits [17,26], fate selection can occur independent of positive feedback or cooperativity in HIV.

### Tat slows promoter toggling by inhibiting LTR ON-to-OFF transitions, leading to bimodality

To understand the molecular mechanisms enabling LTR bimodality in the absence of feedback, we used a validated computational model of HIV [27] and adapted it to an open-loop system where Tat would either modulate (1) burst frequency alone, k_ON_ modulation; (2) burst frequency and burst size, k_OFF_ modulation; or (3) burst size alone by affecting transcriptional efficiency, α modulation (top of Fig 2C, S1–S3 Tables and S1 Data). To model Tat modulation of k_OFF_ alone, a third promoter state, termed Tat-LTR_ON_, was added such that it maintained the same transcriptional efficiency, α, as the LTR_ON_ state. Thus, the transactivated LTR promoter must first transition from Tat-LTR_ON_ to LTR_ON_ and only then can it transition from LTR_ON_ to LTR_OFF_ and fully turn off. This third promoter state, Tat-LTR_ON_, is necessary to generate changes in burst sizes without altering transcriptional efficiency or toggling from the LTR_OFF_ to LTR_ON_ state. The model results are consistent with previous findings that bimodality is not induced through frequency modulation of the LTR (i.e., k_ON_ modulation) or increases in burst size through transcriptional efficiency, α [24,36,37]. However, the model shows that slowing toggling kinetics, or increasing the dwell time in the LTR_ON_ and LTR_TatON_ states (i.e., k_OFF_ modulation), is required for bimodality, and if Tat only affects a single parameter, k_OFF_ modulation is necessary and sufficient (bottom of Fig 2C, S6 Fig and S1 Data).

The interpretation of these results is that, while natural LTR promoter toggling is too quick to generate large enough expression fluctuations for bimodality, Tat transactivation is able to slow the kinetics of toggling, expanding the bimodal regime (Fig 1). The slowing of toggling kinetics reinforces the findings that Tat stabilizes transient pulses of expression from LTR fluctuations [40], by effectively reducing k_OFF_. If Tat does stabilize pulses of expression to control gene-expression variability, then the prediction is that altering Tat-feedback strength would, similar to the open-loop system, control the shape of the gene-expression distribution and bimodality.

### Positive-feedback strength controls whether the expression distribution is unimodal or bimodal in HIV

**Fig 3:**
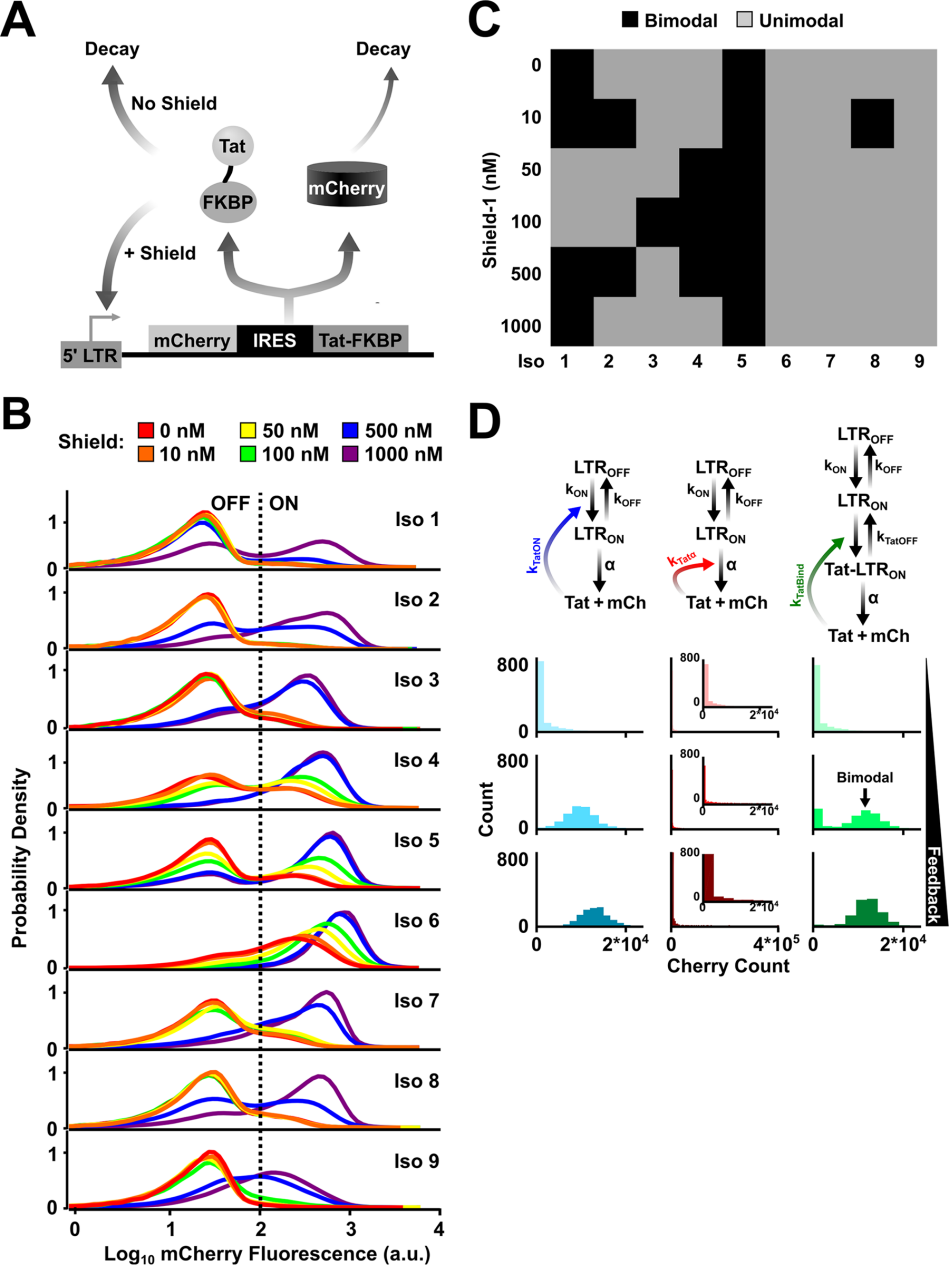
Positive-feedback strength controls whether the expression distribution is unimodal or bimodal in HIV. (A) Schematic of the LTR-mCherry-IRES-Tat-FKBP closed-loop, positive-feedback circuit. The transactivator of transcription (Tat) stability is tuned through the addition of Shield-1 to alter Tat feedback strength (i.e., loop transmission) (S9 Fig). (B) Flow cytometry histograms showing bimodal distribution for 9 isoclonal cell lines exposed to various concentrations of Shield-1. A fraction of isoclones can naturally generate bimodal distributions with low feedback strength (e.g., red [0 nM Shield-1]), but with intermediate positive-feedback strength, bimodal distributions are more prevalent (e.g., green [100 nM Shield-1] or blue [500 nM Shield-1]). The “ON”/ “OFF” threshold was set based on the background level of expression from a naïve Jurkat cell line. (C) Measurement of bimodality for each Shield-1 condition for each isoclonal population in (B) as quantified by the Hartigan Dip Test. The results agree with another metric for measuring bimodality (S7 Fig and S30 Data). Gray squares are determined to be unimodal, and black squares are bimodal. (D) A closed-loop stochastic model (in contrast to the open-loop model in Fig 2C) of long terminal repeat (LTR) promoter toggling that incorporates Tat positive feedback through 1 of 3 alternate mechanisms (Fig 2C). Note that for Tat modulation of k_OFF_ (right), both the LTR_ON_ state and the Tat-LTR_ON_ state produce mCh and Tat at the same rate, α, as described for Fig 2C. The steady-state results for 1,000 simulation runs (modeled for 1,000 hours) show that Tat inhibition of promoter turnoff is sufficient to generate bimodalities (right column, middle panel), whereas alternate Tat positive-feedback mechanisms are unable to generate bimodality in the requisite parameter regimes (S26 Data).

To test the prediction that Tat-feedback strength shapes the expression distribution, we used a synthetic Tat circuit [27] where positive-feedback strength could be manipulated pharmacologically by the addition of a small-molecule, Shield-1, that stabilizes Tat proteolysis (Fig 3A). In this system, a subset of isoclonal cell populations carrying this synthetic circuit naturally generate bimodal distributions (Fig 3B and S25–S29 Data). These clonal differences are mainly due to the genomic location of HIV integration, which can dictate the transcriptional bursting parameters, and the effectiveness of Tat transactivation [24,36]. Though the differences in Tat transactivation potential are not clear, transcriptional parameters of the LTR in the absence of feedback vary due to promoter methylation status, nucleosome acetylation and methylation state, or gene-proximity dependencies [41]. When positive-feedback strength is increased, a significant fraction of the cells generate bimodal distributions and even convert from a unimodal (low peak) into a bimodal (low and high peak) distribution or from a bimodal (low and high peak) to a unimodal (high peak) distribution (Fig 3B, S7 Fig and S25–S30 Data).

Importantly, simulations of Tat positive-feedback circuitry corroborate this phenomenon of bimodal expression at intermediate feedback strength if Tat acts by decelerating LTR toggling kinetics (Fig 3C and S26 Data), in agreement with simulations of the open-loop circuit (Fig 2). Thus, these simulations indicate that Tat-feedback strength likely alters the natural LTR toggling kinetics set by the local integration site [42] to control HIV bimodal-expression patterns. To test if Tat feedback in fact extends pulses of expression (i.e., effective *k*_OFF_ reduction), HIV gene-expression was activated to a high-expression state, using tumor necrosis factor alpha (TNFα), and the circuit was then allowed to relax back to the unperturbed state under varying feedback strengths. TNFα enhances HIV expression by stimulating recruitment of a p50-RelA heterodimer to nuclear factor kappa-light-chain-enhancer of activated B cells (NFkB) binding sites within the LTR [42]. The cells were exposed to TNFα for 24 hours and then allowed to relax back in the presence of strong or weak feedback (S8 Fig). The results show that increasing feedback strength, by dosing cells with increasing amounts of Shield-1, increases the transient in the expressive states, leading to slower transitions from ON to OFF states (S8 Fig and S31 Data), which corroborates previous findings [27,40]. Thus, relaxation to various baseline states is dictated by feedback acting on promoter toggling.

One simplifying assumption in the model is that Tat only modulates a single bursting parameter. To test how relaxing this assumption affects bimodal generation, new simulations in which Tat could modulate multiple bursting parameters were performed. The models allow Tat to alter both burst size and frequency through k_ON_ and k_OFF_, k_ON_ and α, or k_OFF_ and α modulation (S9 Fig). Interestingly, the simulations show that any combination of parameters could yield bimodality (S9 Fig). In each scenario, Tat positive feedback yields nonexponentially distributed “OFF” times and slows toggling kinetics. This result is in agreement with the previous findings that slowing promoter toggling kinetics yields bimodal distributions (Figs 1–3 and S6 Fig).

A few alternate explanations are possible for the observed bimodality. The first is that the bimodality may arise from deterministic cell-to-cell variability [43] where the transcriptional parameters vary between cells, leading to bimodality. However, these minimal circuits display a high level of ergodicity [24,40], suggesting the cell-to-cell variability in the transcriptional parameters is minimal. Second, HIV feedback may be bistable (i.e., exist in 1 of 2 stable states [high or low] [17]). Bimodality observed from bistable circuits results from fluctuations around latching feedback strengths (S11 Fig). Previous studies analyzing fluctuations in noise to measure feedback strength, cooperativity in feedback, or stability of the “ON” state found that HIV feedback lacks the canonical features of bistability [34,35,40]. Last of all, HIV feedback may latch, meaning small increases in Tat would be drastically amplified to saturable levels upon which the system would then latch in a high state. Note that the latching behavior can be present in deterministically monostable feedback [40]. To test this, here, we directly quantified the feedback strength—to test if the feedback-induced bimodality results from latching feedback—by use of the small-signal loop gain, a direct measure of feedback strength [40,44,45]. The small-signal loop gain was quantified by measuring changes in LTR expression associated with changing Tat stability (S11 Fig) or increasing Tat concentration (S12 Fig and S32 Data). First, we verified that green fluorescent protein (GFP) fluorescence intensity was linearly correlated to GFP-protein abundance, as shown [32,46], by quantifying the fluorescence intensity of known concentrations of soluble GFP by microscopy and then comparing these values to the GFP fluorescence intensity of the LTR-GFP-IRES-Tat-FKBP circuit in 2 isoclonal populations when feedback was either inactive or active (S10 Fig and S33 and S34 Data). As expected, the GFP fluorescence intensity was well within the linear GFP-protein concentration regime for both microscopy and flow cytometry (S10 Fig and S33 and S34 Data). After verifying that fluorescence intensity scales linearly with protein abundance, we used fluorescence intensity to quantify changes in protein expression associated with altering Tat stability or Tat concentration. In agreement with other measures of HIV feedback strength [40], we find that Tat positive feedback appears to be nonlatching (S11 and S12 Figs and S32 Data). Interestingly, unlike systems that latch, nonlatching feedback strength inherently renders the system relatively insensitive to small fluctuations [47] (i.e., HIV will not drastically change expression profile or latch in response to a small fluctuation) lending a molecular explanation for the insensitivity of HIV circuitry to external cues [48,49].

### HIV Tat positive feedback appears optimized to robustly generate bimodal distributions

**Fig 4:**
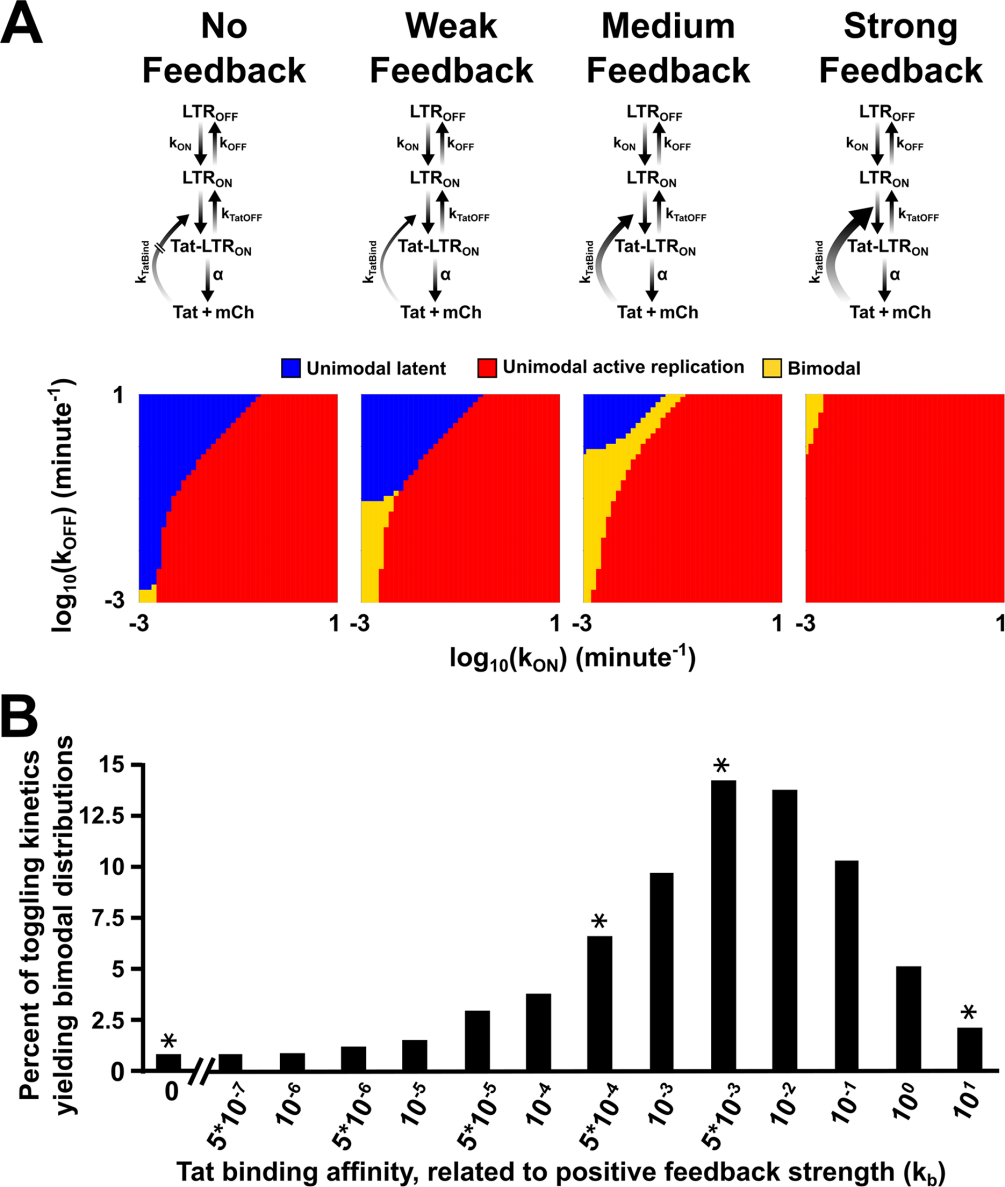
HIV positive feedback appears optimized to robustly generate bimodal distributions. (A) Varying the positive-feedback strength changes the toggling kinetics to yield a larger regime for bimodality within the physiological parameter range. The results for the parameter scans are shown for “No Feedback” (left) and increasing feedback strengths. Whether a population was unimodal latent (blue), unimodal active replication (red), or bimodal (orange) was determined for each set of parameters as described in the Materials and methods section. For the modality analysis, each mode was required to contain at least 0.1% of the population. (B) The percent of toggling kinetics that yield bimodal distributions for varying feedback strengths. The asterisks above the bars represent the feedback strengths shown in (A) (S35 Data).

The combination of nonlatching feedback coupled to a toggling promoter allows for bimodal generation across a wide range of Tat concentrations (Fig 2) and feedback strengths (Fig 3). Promoters driving nonlatching feedback can exhibit extended, transient pulses of expression before reverting back to the initial system state [8]. To test if this mechanism of extended-duration transient pulses was responsible for generating bimodality in the LTR, we built a specific model of the LTR to map out the phase space of feedback strengths that would allow for LTR bimodality given the known toggling parameters (S1 Table). The model specifically considers promoter toggling coupled to weak positive feedback and examined the effect of changing feedback strength (from weak nonlatching to strong nonlatching). In agreement with previous theoretical predictions [19,20], intrinsic slow promoter toggling is sufficient to generate bimodality, but only in a very narrow parameter regime (Figs 1 and 4A).

To explore if weak nonlatching positive feedback might explain the robust generation of bimodality that was experimentally observed, we incorporated dose-response data from the open-loop circuit into the model and generated an input-output function (S13 Fig) to quantify the relationship between Tat and *k*_OFF_ values. This approach allows the open-loop data to be mapped onto a model containing feedback (Fig 4A). The output of the resulting model shows a striking dependence of bimodality on feedback strength (Fig 4B and S31 Data). Specifically, as feedback strength increases from zero, the bimodality regime significantly expands. However, as feedback increases further, to strong nonlatching feedback strengths, there is a drastic reduction in the potential for bimodal generation (Fig 4B, S14 Fig and S35 Data). This acute contraction of the bimodal regime likely results from drastic amplifications of small noise spikes that drive the system to stay on [17]. Interestingly, the model predicts that bimodality is generated across approximately 13% of the parameter values for the HIV system (Fig 4B and S35 Data), in agreement with experimentally observed frequencies for spontaneous bimodal generation across the HIV-integration landscape [32]. Thus, HIV’s moderate feedback strength (S11 and S12 Figs and S32 Data) appears optimized to slow promoter-toggling kinetics into the regime that enables bimodality.

### Robust bimodality results from positive feedback decoupling expression noise from mean levels

**Fig 5:**
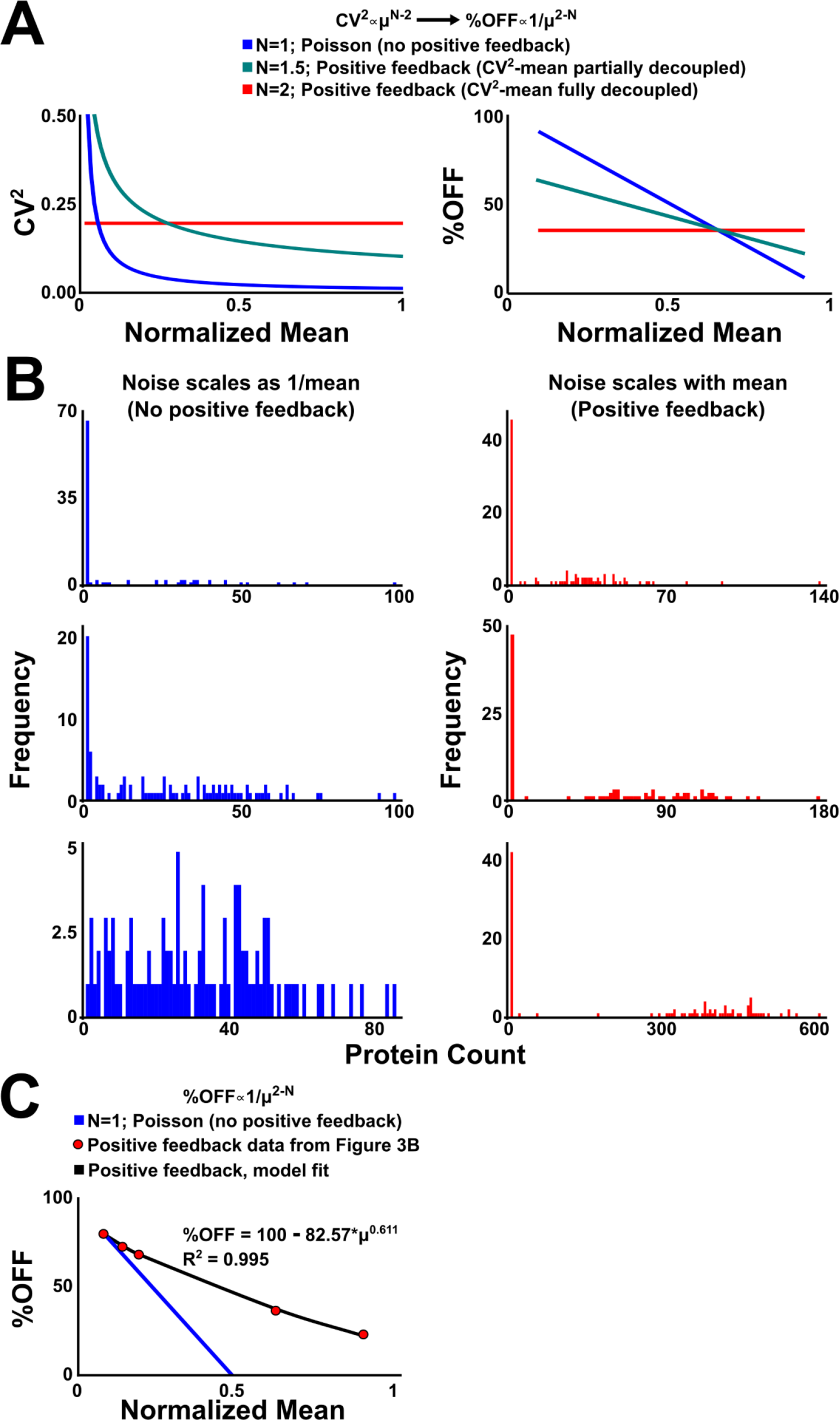
Nonlatching positive feedback substantially dampens the Poissonian noise-mean inverse relationship, allowing stochastic extinction despite increasing mean-expression levels. (A) In the classical Poisson or super-Poissonian transcriptional burst models [50], the expression mean scales with variance (σ^2^ ∝ μ) such that the noise magnitude (CV^2^ = σ^2^ / μ^2^) decreases proportionally to the inverse of the mean. Nonlatching positive feedback breaks the Poissonian relationship such that σ^2^ ∝ μ^N^ with 1 < *N* < 2 [44]. In the extreme case where *N* = 2, CV^2^ becomes independent of the mean. (B) Monte-Carlo (Gillespie) simulations for three different population mean values in absence (left) and presence (right) of positive feedback showing that stochastic extinction can be decoupled from the mean (when *N* = 2). (C) Analysis of the data in Fig 3 shows that the Tat circuit displays partial decoupling of noise and mean (*N* ≈ 1.5).

Since the circuit’s bimodality is ultimately dependent upon fluctuation-driven (i.e., stochastic) extinction of Tat, we next sought to determine how increasing expression levels influenced bimodality. In the classical Poisson or super-Poissonian transcriptional burst models [50], the expression mean scales with variance (σ^2^ ∝ μ) such that the noise magnitude (CV^2^ = σ^2^ / μ^2^) decreases proportionally to the inverse of the mean squared (Fig 5A) and the extinction probability can be shown to be as follows (S1 Text):

$$Prob_{extinct}=\int_{-\infty }^{0}\frac{1}{\sqrt{2\pi \sigma^{2}}}e^{-\frac{{\left(P-\mu \right)}^{2}}{2\sigma^{2}}}dP$$ (1)

However, nonlatching positive feedback breaks the Poissonian relationship such that σ^2^ ∝ μ^N^ with 1 < *N* < 2 [44]. In the extreme case where *N* = 2, CV^2^ becomes independent of the mean and the extinction probability becomes the following (S1 Text):

$$Prob_{extinct}=\int_{-\infty }^{0}\frac{1}{\sqrt{2\pi \sigma^{2}{}_{NFB}}}e^{-\frac{{\left(x-\mu_{NFB}\right)}^{2}}{2\sigma^{2}{}_{NFB}}}dx,$$ (2)

where *σ*^*2*^_*NFB*_ and *μ*_*NFB*_ are the variance and the mean for the nonfeedback case, respectively. Importantly, Eq 2 shows that stochastic extinction can be decoupled from the mean (when *N* = 2), and simulations verified that such perfect decoupling was possible (Fig 5B). Analysis of the experimental data in Fig 3 shows that the Tat circuit displays partial decoupling of noise and mean with *N* ≈ 1.5 (Fig 5C). Thus, Tat circuitry enables greater stochastic extinction over a broader range than other circuitries (e.g., no feedback or latching positive feedback) would be able to achieve.

## Discussion

In summary, HIV’s Tat circuit seems particularly well suited for generating bimodal expression patterns, and alternate single-parameter mechanisms for Tat function (e.g., increasing burst frequency alone rather than slowing toggling kinetics) appear to severely limit or completely abrogate the potential for bimodality. The precise architecture of this robust bimodal-generator circuit in such a rapidly adapting virus suggests that bimodality in HIV expression (i.e., latent and active replication modes) may be a beneficial trait that has been selectively maintained [38]. In contrast with other known roles for positive feedback (e.g., bistability and noise amplification), these findings demonstrate a further role for positive feedback as a mechanism for robust generation of bimodality [51]. On a conceptual level, this ability of positive feedback to expand the bimodal regime into physiological ranges may be related to positive feedback’s ability to expand the regime where sustained oscillations occur [52,53]. Consequently, positive-feedback circuits may have evolved not only for signal amplification but also to stabilize certain dynamic phenotypes (e.g., bimodality and oscillations) in diverse biological systems.

From a basic HIV biology standpoint, these results on Tat’s mechanism of action may have therapeutic implications for HIV cure approaches. Specifically, Tat protein addition reactivates HIV latency more potently than current chromatin remodeling latency-reversing agents (LRAs) such as histone deacetylase inhibitors (HDACis) [42]. Despite the known role of Tat as a transcriptional elongation factor, there has been no clear mechanistic explanation as to why Tat protein is more potent than LRAs that are transcriptional activators (e.g., HDACis). Conventional LRAs (e.g., protein kinase C [PKC] agonists and HDACis) only affect k_ON_, and we have previously shown that agents that simultaneously reduce k_OFF_ and k_ON_ potentiate reactivation [54]. Hence, the finding herein that Tat alters k_OFF_, coupled with the magnitude of the Tat-induced k_OFF_ change, provides a mechanistic explanation as to why Tat is so effective for latency reversal. The findings also suggest that Tat-based strategies and conventional LRA strategies could be used synergistically, and new approaches aimed at simultaneously reducing k_OFF_ and increasing k_ON_ would be optimal for “shock-and-kill” strategies, while conversely increasing k_OFF_ and decreasing k_ON_ would be optimal for “block-and-lock” strategies.

## Materials and methods

### Molecular cloning procedures

The sequence of Tat from recombinant clone pNL4-3, GenBank: AAA44985.1, M19921, was used. To clone the LTR-mCherry-IRES-Tat-FKBP construct, d2GFP was swapped with mCherry using BamHI and EcoRI restriction sites [27]. To clone the Tet-Tat-Dendra-FKBP plasmids, Tat-Dendra or Tet-Tat-Dendra was swapped with YFP-Pif from the pHR-TREp-YFP-Pif plasmid (a gift from Wendell Lim’s laboratory at UCSF) using BamHI and NotI restriction sites. The full-length virus was generated as described [27].

### Preparation of the GFP standard curve

For the GFP standard curve, a stock solution of 1 g/L (= 30.58 μM) recombinant eGFP (Cell Biolabs) was diluted 500-, 1,000-, 5,000-, and 10,000-fold (= 61.12, 30.58, 6.11, and 3.06 nM, respectively). These soluble GFP standards of known concentration were imaged in an 8-well chambered imaging dish using the same confocal microscope settings as subsequent cellular GFP imaging.

### Cellular GFP imaging

Isoclonal populations were incubated with shield for 20 hours (if applicable). Approximately 6 x 10^5^ cells were washed with 2 mL of PBS solution and then immobilized on a Cell-Tak (Fisher) coated 8-well chambered imaging dish, using the manufacturer’s protocol. Both soluble GFP standards and cellular GFP were imaged on a Nikon Ti-E microscope equipped with a W1 Spinning Disk unit, an Andor iXon Ultra DU888 1k x 1k EMCCD camera, and a Plan Apo VC 100x/1.4 oil objective in the UCSF Nikon Imaging Center; the exposure time was 500 ms with 50% laser power. Approximately 15 xy locations were randomly selected for each isoclonal population. After background and autofluorescence subtraction from the cellular GFP images, the cellular GFP concentration was determined from the GFP standard curve. The cellular volume was approximated from the measured cellular dimensions, assuming a spherically shaped cell.

### Recombinant virus production and infections

Lentivirus was generated in 293T cells and isolated as described [32,55]. To generate the isoclonal closed-loop circuit populations, lentivirus was added to Jurkat T Lymphocytes at a low MOI to ensure a single integrated copy of proviral DNA in the infected cells. The cells were stimulated with TNFα and Shield-1 for 18 hours before sorting for mCherry. Isoclonal and polyclonal populations were created as described [32]. The sorting and analysis of the cells infected was performed on a FACSAria II. Inducible-Tat cells were generated by transducing Jurkat cells with Tet-Tat-Dendra-FKBP and SFFV-rTta lentivirus at high MOI [27]. The cells were incubated in Dox for 24 hours and then FACS sorted for Dendra+ cells to create a polyclonal population. To create the Tet-Tat-Dendra-FKBP + LTR-mCherry cells, the polyclonal population was infected with LTR-mCherry lentivirus at a low MOI. Before sorting for mCherry+ and Dendra+ cells, Dox was added at 500 ng/mL for 24 hours, and single cells were FACS sorted and expanded to isolate isoclonal populations.

### Flow cytometry analysis

Flow cytometry data were collected on a BD FACSCalibur DxP8, BD LSR II, or HTFC Intellicyt for stably transduced lines and sorting. Flow cytometry data were analyzed in FlowJo (Treestar, Ashland, Oregon, United States) and using customized MATLAB code [27].

### Mathematical model and stochastic simulations

A simplified 2-state model of LTR toggling and Tat positive feedback was constructed based on experimental data of LTR toggling [24,36] and simulated using the Gillespie algorithm [56] in MATLAB to test how altering toggling kinetics and feedback strength would affect the activity of the circuit. At least 1,000 simulations were run for each condition.

Alternatively, to sweep the parameter space of different modulations of the Tat circuit, the accurate chemical master equation (ACME) method [57,58] was used to directly solve the chemical master equation (CME) to obtain the full probability landscapes of protein copy number. For each parameter pair in the sweeping, the protein probability landscape was computed at day 3 or at steady state. The phenotype of bimodality or unimodality at different parameter pairs was based on the numbers and locations of probability peaks in the landscape using the bimodality analysis approach described in the Materials and methods section.

### Bimodal analysis

Two approaches were taken to quantify whether a distribution from the experimental data or simulations was bimodal or unimodal. The first, applied to both simulations and experimental data, was to convert the fluorescence density data using the *bkde* function in the KernSmooth package in R to a binned kernel density [59]: the KernSmooth R package is available at https://cran.r-project.org/web/packages/KernSmooth/index.html. To filter out biologically irrelevant noise in the data, the data points with fluorescence density less than 1 or small peaks lower than 0.05 in calculated kernel density function were ignored. The number of modality peaks was determined by calculating the second-order derivative of the kernel density. The second approach, only applied to the experimental data, was to utilize the Hartigan Dip Test, a dip statistic that can test for multimodality by testing for maximal differences and ascertain the probability that a particular distribution is unimodal [39]. Code for the Hartigan Dip Test was obtained from http://nicprice.net/diptest/, adapted from Hartigan’s original Fortran Code for MATLAB.

## Supporting information

S1 Data Experimental setup for Fig 2B and S2, S3, S12 and S13 Figs and the raw numbers for Fig 2C. The excel spreadsheet has multiple pages. The first page provides the experimental setup for Fig 2B and S2, S3, S12 and S13 Figs. The setup also explains which of the flow cytometry (.fcs) files in S3–S11 Data correspond to each condition. The second through the fourth pages provide the raw numbers used to generate Fig 2C from simulations as explained in the Materials and methods section.
(XLSX)

S2 Data Tables of the raw numbers of the Hartigan Dip Test corresponding to Fig 2B. The file (.mat format) gives the Hartigan Dip Test value corresponding to each condition in S3–S11 Data. The threshold of *p* < 0.3 was used to generate Fig 2B.
(MAT)

S3 Data Flow cytometry files for Isoclone 1 of the open-loop system. The files are labeled according to the experimental setup on the first sheet of S1 Data. The corresponding mCherry and Dendra fluorescence values (see S12 Data) were taken according to the gating strategy in S21 Data and were used to generate the numbers in Fig 2B and S2, S3, S12 and S13 Figs.
(ZIP)

S4 Data Flow cytometry files for Isoclone 2 of the open-loop system. The files are labeled according to the experimental setup on the first sheet of S1 Data. The corresponding mCherry and Dendra fluorescence values (see S13 Data) were taken according to the gating strategy in S21 Data and were used to generate the numbers in Fig 2B and S2, S3, S12 and S13 Figs.
(ZIP)

S5 Data Flow cytometry files for Isoclone 3 of the open-loop system. The files are labeled according to the experimental setup on the first sheet of S1 Data. The corresponding mCherry and Dendra fluorescence values (see S14 Data) were taken according to the gating strategy in S21 Data and were used to generate the numbers in Fig 2B and S2, S3, S12 and S13 Figs.
(ZIP)

S6 Data Flow cytometry files for Isoclone 4 of the open-loop system. The files are labeled according to the experimental setup on the first sheet of S1 Data. The corresponding mCherry and Dendra fluorescence values (see S15 Data) were taken according to the gating strategy in S21 Data and were used to generate the numbers in Fig 2B and S2, S3, S12 and S13 Figs.
(ZIP)

S7 Data Flow cytometry files for Isoclone 5 of the open-loop system. The files are labeled according to the experimental setup on the first sheet of S1 Data. The corresponding mCherry and Dendra fluorescence values (see S16 Data) were taken according to the gating strategy in S21 Data and were used to generate the numbers in Fig 2B and S2, S3, S12 and S13 Figs.
(ZIP)

S8 Data Flow cytometry files for Isoclone 6 of the open-loop system. The files are labeled according to the experimental setup on the first sheet of S1 Data. The corresponding mCherry and Dendra fluorescence values (see S17 Data) were taken according to the gating strategy in S21 Data and were used to generate the numbers in Fig 2B and S2, S3, S12 and S13 Figs.
(ZIP)

S9 Data Flow cytometry files for Isoclone 7 of the open-loop system. The files are labeled according to the experimental setup on the first sheet of S1 Data. The corresponding mCherry and Dendra fluorescence values (see S18 Data) were taken according to the gating strategy in S21 Data and were used to generate the numbers in Fig 2B and S2, S3, S12 and S13 Figs.
(ZIP)

S10 Data Flow cytometry files for Isoclone 8 of the open-loop system. The files are labeled according to the experimental setup on the first sheet of S1 Data. The corresponding mCherry and Dendra fluorescence values (see S19 Data) were taken according to the gating strategy in S21 Data and were used to generate the numbers in Fig 2B and S2, S3, S12 and S13 Figs.
(ZIP)

S11 Data Flow cytometry files for Isoclone 9 of the open-loop system. The files are labeled according to the experimental setup on the first sheet of S1 Data. The corresponding mCherry and Dendra fluorescence values (see S20 Data) were taken according to the gating strategy in S21 Data and were used to generate the numbers in Fig 2B and S2, S3, S12 and S13 Figs.
(ZIP)

S12 Data Raw numbers of mCherry and Dendra fluorescence values for Isoclone 1 of the open-loop system. The corresponding mCherry and Dendra fluorescence values were taken according to the gating strategy in S21 Data applied to the flow cytometry files (S3 Data) and were used to generate the numbers in Fig 2B and S2, S3, S12 and S13 Figs.
(MAT)

S13 Data Raw numbers of mCherry and Dendra fluorescence values for Isoclone 2 of the open-loop system. The corresponding mCherry and Dendra fluorescence values were taken according to the gating strategy in S21 Data applied to the flow cytometry files (S4 Data) and were used to generate the numbers in Fig 2B and S2, S3, S12 and S13 Figs.
(MAT)

S14 Data Raw numbers of mCherry and Dendra fluorescence values for Isoclone 3 of the open-loop system. The corresponding mCherry and Dendra fluorescence values were taken according to the gating strategy in S21 Data applied to the flow cytometry files (S5 Data) and were used to generate the numbers in Fig 2B and S2, S3, S12 and S13 Fig.
(MAT)

S15 Data Raw numbers of mCherry and Dendra fluorescence values for Isoclone 4 of the open-loop system. The corresponding mCherry and Dendra fluorescence values were taken according to the gating strategy in S21 Data applied to the flow cytometry files (S6 Data) and were used to generate the numbers in Fig 2B and S2, S3, S12 and S13 Figs.
(MAT)

S16 Data Raw numbers of mCherry and Dendra fluorescence values for Isoclone 5 of the open-loop system. The corresponding mCherry and Dendra fluorescence values were taken according to the gating strategy in S21 Data applied to the flow cytometry files (S7 Data) and were used to generate the numbers in Fig 2B and S2, S3, S12 and S13 Figs.
(MAT)

S17 Data Raw numbers of mCherry and Dendra fluorescence values for Isoclone 6 of the open-loop system. The corresponding mCherry and Dendra fluorescence values were taken according to the gating strategy in S21 Data applied to the flow cytometry files (S8 Data) and were used to generate the numbers in Fig 2B and S2, S3, S12 and S13 Figs.
(MAT)

S18 Data Raw numbers of mCherry and Dendra fluorescence values for Isoclone 7 of the open-loop system. The corresponding mCherry and Dendra fluorescence values were taken according to the gating strategy in S21 Data applied to the flow cytometry files (S9 Data) and were used to generate the numbers in Fig 2B and S2, S3, S12 and S13 Figs.
(MAT)

S19 Data Raw numbers of mCherry and Dendra fluorescence values for Isoclone 8 of the open-loop system. The corresponding mCherry and Dendra fluorescence values were taken according to the gating strategy in S21 Data applied to the flow cytometry files (S10 Data) and were used to generate the numbers in Fig 2B and S2, S3, S12 and S13 Figs.
(MAT)

S20 Data Raw numbers of mCherry and Dendra fluorescence values for Isoclone 9 of the open-loop system. The corresponding mCherry and Dendra fluorescence values were taken according to the gating strategy in S21 Data applied to the flow cytometry files (S11 Data) and were used to generate the numbers in Fig 2B and S2, S3, S12 and S13 Figs.
(MAT)

S21 Data Gating strategy on flow cytometry data for isoclonal open-loop populations. The forward-scatter and side-scatter values were used to determine the live population (top left). The live population was gated on green fluorescent protein (GFP) values over the axis (top right) to then quantify the mCherry values (bottom left) and GFP values (bottom right). The gating strategy in S21 Data applied to the flow cytometry files (S10 Data) and was used to generate the numbers in Fig 2B and S2, S3, S12 and S13 Figs.
(PDF)

S22 Data Bimodal test raw values for the open-loop system. This file presents the binary values for determining whether an experimental condition in 1 of the open-loop populations (S3 Fig) is bimodal according to the bimodality test described in the Materials and methods section.
(XLSX)

S23 Data Gating strategy for the Ld2GITF (positive feedback loop expressing GFP) population. The forward-scatter and side-scatter values were used to determine the live population (left). The live population green fluorescent protein (GFP) values were used to generate the data in S8 and S10 Figs.
(PDF)

S24 Data Raw values of the Cherry and Dendra signal from single-cell time-lapse microscopy data in S4 Fig. The file contains each individual cell’s signal for mCherry and Dendra over time and the mean values for each condition in S4 Fig (all contained in a.mat file).
(MAT)

S25 Data Experimental setup and Hartigan Dip Test *p*-values for the closed-loop system in Fig 3 and S7 and S11 Figs. The excel file explains which flow cytometry file in S27 Data corresponds to each isoclone and condition in the closed-loop system used in Fig 3. The second sheet of the file gives the raw *p*-values of the Hartigan Dip Test for each condition of each isoclone and the TRUE/FALSE for whether the value is <0.3.
(XLSX)

S26 Data Raw numbers used to generate Fig 3D. The excel sheet gives the raw numbers used to generate the graphs in Fig 3D for different modes of action and different feedback strengths of Tat used in the simulations.
(XLSX)

S27 Data Flow cytometry files for all of the closed-loop isoclonal populations in Fig 3. The file labels in conjunction with S25 Data can be used to track which file corresponds to which condition and isoclonal population. The gating strategy is found in S28 Data, and the raw numbers extracted from the files used to generate Fig 3B and 3C and S7 and S11 Figs can be found in S29 Data.
(ZIP)

S28 Data Gating strategy for the LChITF (positive feedback loop expressing mCherry) isoclonal populations. The forward-scatter and side-scatter values were used to determine the live population (left). The live mCherry population was then gated to remove debris that fluoresced at the axis (middle graph), and the mCherry values were extracted. These values were used in Fig 3B and 3C and S7 and S11 Figs and can be found in S29 Data.
(PDF)

S29 Data Raw numbers of mCherry for the isoclonal closed-loop populations. The corresponding mCherry fluorescence values were taken according to the gating strategy in S28 Data applied to the flow cytometry files (S27 Data) and were used to generate the numbers in Fig 3B and 3C and S7 and S11 Figs.
(ZIP)

S30 Data Bimodal test raw values for the closed-loop system. This file presents the binary values for determining whether an experimental condition in 1 of the closed-loop populations (S7 Fig) is bimodal according to the bimodality test described in the Materials and methods section.
(XLSX)

S31 Data Raw values for the data in S8 Fig. This file contains the raw numbers used to generate S8 Fig according to the gating strategy described in S23 Data.
(XLSX)

S32 Data Raw values for the data in S12 Fig. This file contains the raw numbers used to generate S12 Fig according to the gating strategy described in S21 Data and the files and raw numbers extracted from S3–S20 Data.
(XLSX)

S33 Data Flow cytometry files corresponding to S10 Fig. The raw numbers were extracted from these flow cytometry files to generate S10 Fig according to the gating strategy described in S23 Data.
(ZIP)

S34 Data Raw values for the data in S10 Fig. The raw numbers were extracted from the flow cytometry files in S33 Data according to the gating strategy described in S23 Data. These data were used to show the linear relationship between fluorescence intensity and protein numbers that allows for quantification of the small-signal gain in S11 and S12 Figs.
(XLSX)

S35 Data Raw values quantifying the amount of bimodality observed in the simulations in Fig 4 and S14 Fig according to various cutoffs. The excel file contains the raw numbers for how many of the simulation parameters yield bimodality according to various cutoffs. FB stands for feedback strength, and the percent is the implied cutoff.
(XLSX)

S1 Fig Promoter toggling kinetics control the separation of gene-expression peaks due to transient production and decay. For a given time, the rate of switching between the ON and OFF promoter states (top pulse trains) is related to the duration of time in a specific promoter state. The duration of the promoter state determines the length, or separation from the mean (cyan line, same value for each panel), of the transient production or decay of gene-expression products. Increasing promoter kinetics reduces transients and the separation between potential peaks in a bursty system (top left moving to the right and then bottom left moving to the right).
(TIF)

S2 Fig The long terminal repeat (LTR) produces bimodal distributions in response to unimodal Tat inputs. (A) Histograms of the transactivator of transcription (Tat) input to the LTR, as measured by Dendra fluorescent signal, are unimodal across all combinations of doxycycline and Shield-1. The colors of the lines indicate increasing doxycycline concentrations (red, 0 ng/mL → orange, 2.5 ng/mL → yellow, 5 ng/mL → green, 12.5 ng/mL → cyan, 25 ng/mL → blue, 50 ng/mL → pink, 250 ng/mL → magenta, 500 ng/mL), and the increasing brightness of the same color represents increasing Shield-1 concentrations (0, 10, 50, 100, 500, and 1,000 nM). (B) Histograms of LTR output as measured by mCherry fluorescent signal. The “Dim”/“Bright” threshold was set based on each population’s mCherry expression in the absence of doxycycline or Shield-1 (i.e., no Tat). The change in signal in the Bright population was used to determine the small-signal loop gain (S12 Fig) in response to Tat. The graphs were generated by ks-density clustering of the data, which can smooth features of a rough distribution, exaggerating particular features. Noticeably, some of the seemingly bimodal distributions do not pass the quantitative metrics used in Figs 2 and 3 and S3 and S7 Figs (S1–S24 Data).
(TIF)

S3 Fig Bimodality analysis for the open-loop system. Nine isolconal populations of the open-loop circuits described in Fig 2 were exposed to 48 different doxycycline or Shield-1 concentrations. The populations were assessed for the number of modes as described in the Materials and methods section. Briefly, fluorescence intensity data were smoothed using the *bkde* function in the KernSmooth package in R to a binned kernel density. The number of modality peaks was calculated by taking the second-order derivative of the kernel density. Gray squares are unimodal, and black squares are bimodal (S22 and S23 Data).
(TIF)

S4 Fig Tat activation of the long terminal repeat (LTR) controls expression pulses. Single-cell time-lapse fluorescence microscopy of the open-loop circuit without doxycycline (black lines) or with 25 ng/mL (red lines), 100 ng/mL (cyan lines), or 500 ng/mL (green lines) of doxycycline. Both Dendra (i.e., transactivator of transcription (Tat) levels) and mCherry (i.e., LTR activity) fluorescence levels were tracked over time. Variable Tat inputs as measured by Dendra fluorescence lead to variable expression pulses from the LTR as measured by Cherry expression (S24 Data).
(TIF)

S5 Fig Full-length HIV decision-making can be controlled in the absence of feedback or cooperativity. Schematic of the full-length HIV open-loop circuit (top). Doxycycline addition induces transactivator of transcription (Tat) expression, which can activate expression of the full-length HIV virus with a fluorescent mCherry reporter. Cells were initially infected in the absence (red histogram) or presence (blue histogram) of doxycycline, and a time point was taken 24 hours post infection (left side, “Initial Infection”). Doxycycline was then added to a split of the “No Dox” at the Initial Infection for 24 hours to look for HIV reaction (right side, “Latent Reactivation”).
(TIF)

S6 Fig The fluctuations in mCherry depend on the mechanism of transactivator of transcrtiption (Tat) transactivation. (A) Each parameter set was allowed to run for 1,000 stochastic simulations, where Tat would work through k_ON_ (green), k_OFF_ (pink), or alpha (black lined) alone. The average protein count is equivalent for all the simulations. (B) The time course of the mCherry count over time, showing the extent of stochastic fluctuations when Tat affects k_ON_, k_OFF_, or alpha. Three representative traces are shown for each (S1 Data).
(TIF)

S7 Fig Bimodality analysis for the closed-loop feedback system. Nine isoclonal populations were exposed to various concentrations of Shield-1 as described in Fig 3. The number of modes was determined as described in the Materials and methods section. Briefly, fluorescence intensity data were smoothed using the *bkde* function in the KernSmooth package in R to a binned kernel density. The number of modality peaks was calculated by taking the second-order derivative of the kernel density. Gray squares are unimodal, and black squares are bimodal (S30 Data).
(TIF)

S8 Fig Positive-feedback strength sets the steady-state activity and percentage of cells in an active state. A polyclonal population of Ld2GITF (positive feedback loop expressing GFP) cells were exposed to tumor necrosis factor alpha (TNFα) for 24 hours (−24 to 0 hours), and then the cells were washed and split into 1 cultures, 1 with Shield-1 (1 uM, blue) and 1 in the absence of Shield-1 (0 uM, gray). Green fluorescent protein (GFP) measurements were taken every 24 hours, and the mean fluorescence intensity (right axis) or the percentage of cells in the ON state (left axis) was quantified. In the absence of Shield-1 after 72 hours, the cells returned to the unperturbed state in both percent ON and mean fluorescence intensity. In the presence of Shield-1, positive-feedback strength is increased, and the system remains activated for a longer duration of time. Importantly, both populations return to the state of no TNFα addition, i.e., no bistability (S31 Data).
(TIF)

S9 Fig Simulations of Tat modulating 2 parameters of transcriptional bursting. We consider 3 different phenotypes: unimodality of latency (blue areas), unimodality of activation (red areas), and bimodality (yellow areas). The phase diagrams of phenotypes for 3 different modulations based on the steady-state probability landscapes—k_ON_-k_OFF_ (left graphs), k_ON_-alpha (middle graphs), and k_OFF_-alpha (right graphs)—are shown in part A, and the phenotype phase diagrams based on the day 3 probability landscapes are shown in part B. Details about the models and parameter sweeping can be found in the Materials and methods section. In the modulations of k_ON_-k_OFF_ (left graphs) and k_ON_-alpha (middle graphs), some parameter pairs are bimodal at day 3 (yellow area in part B) but become unimodality of activation at the steady state (red area in part A). This is due to the slow evolution of the probability landscape in these parameter pairs. The phenotypes of all parameter pairs in the k_OFF_-alpha (right graphs) modulation at steady state are consistent with those at day 3. All simulations were started with initial toggling kinetics of k_ON_ = 0.001/min, k_OFF_ = 0.01/min, and the rest of the parameters can be found in S1–S3 Tables.
(TIF)

S10 Fig The fluorescent enhanced green fluorescent protein (eGFP) signal linearly increases with GFP concentration. (A) Enhanced GFP (eGFP) calibration curve; dilutions of soluble recombinant eGFP protein were imaged by confocal microscopy. (B) Confocal microscopy (using the same microscope settings as in panel A) and flow cytometry showing the mean fluorescence intensity for 2 isoclonal populations (Iso 1 and Iso 3) of Jurkat Ld2GITF (positive feedback loop expressing GFP) cells—containing a single integration of the Ld2GITF (LTR-d2GFP-IRES-Tat-FKBP) construct—incubated in the presence (+) and absence (−) of Shield-1 (active or inactive feedback, respectively). The GFP levels fall well within the linear regime found in panel A. (C) Mean flow cytometry GFP intensity compared to mean cellular GFP number (calculated via approximate cellular volume) showing a linear relationship (R^2^ = 0.99) (S33 and S34 Data).
(TIF)

S11 Fig Transactivator of transcription (Tat) positive feedback is nonlatching. (A) A schematic showing the input-output relationship for a positive-feedback loop under the control of a constitutive promoter. Unimodal signal inputs of varying strengths reach a constitutive promoter encoding for a transcription factor (TF), which initiates positive feedback. The level of amplification due to positive feedback is quantified by the small-signal loop gain. For loop gains < 1 across all protein concentrations, the system displays nonlatching feedback and the results in a unimodal output over the abundance regime. However, if small-signal loop gain increases with protein abundance to approximately 1, small input fluctuations are drastically amplified and can generate a bimodal distribution in the output (bottom right). The error bars around the circles in A (right-hand graphs) represent, for a population of cells that receive the same inputs, the fluctuations that would lead some cells to display higher or lower small-signal loop gains. (B) Quantification of the small-signal loop gain of the closed-loop circuit for the 2 isoclonal Ld2GITF populations used in S10 Fig—to verify that Tat feedback is nonlatching in the linear fluorescence-to-protein regime. (C) Quantification of the small-signal loop gain for the closed-loop circuits of the 9 isoclonal LChITF populations used in Fig 3 showing that Tat feedback is nonlatching (S1–S25 Data).
(TIF)

S12 Fig Quantification of the open-loop small-signal loop gain shows nonlatching feedback. (A) Plot of the fold change in transactivator of transcription (Tat)-Dendra abundance versus the fold change in mCherry ON population expression for 9 isoclonal populations. (B) Quantification of the small-signal open-loop gain of the 9 isoclonal populations. These values are representative of the expected small-signal loop gain for an intact circuit with feedback. Importantly, all 9 isoclonal populations indicate that Tat feedback is nonlatching (S32 Data).
(TIF)

S13 Fig The response of the long terminal repeat (LTR) to transactivator of transcription (Tat) is biphasic; the LTR is sensitive to low levels of Tat but insensitive to higher levels of Tat. Plot of normalized LTR-mCherry output to normalized Tat-Dendra fluorescence for the 11 clonal populations (Fig 2). The data are best fit with a logarithmic function but can also be represented with 2 linear fits (R^2^ = 0.98): 1 fit for the sensitive region (between 0 and 0.2, Normalized Tat-Dendra Fluorescence) and 1 fit for the insensitive region (between 0.2 and 1, Normalized Tat-Dendra Fluorescence) (S1–S22 Data).
(TIF)

S14 Fig Nonmonotonic relation between the percent of toggling kinetics that yield bimodality versus feedback strength. To simulate various feedback strengths, the binding affinity, k_b_, was tuned from 5 x 10^−7^ to 10, on a parameter scan across k_ON_ and k_OFF_ values ranging from 0.001 to 10/minute. Next, of those parameter scans, a bimodality test was performed (Materials and methods). The percentage of parameters that yielded bimodality was then quantified. Various thresholds were set to determine whether a population was bimodal by requiring that each mode had to have 10^−8^%, 0.1%, 1%, 5%, or 10% of the total population (S35 Data).
(TIF)

S1 Table Chemical reaction scheme (with parameters) for stochastic simulations of circuits where Tat only modulates k_OFF_. For the open-loop circuit with no positive feedback, the ** reaction is present, but the * reaction is not. The variable input in the ** parameter represents the different transactivator of transcription (Tat) inputs, which experimentally are varied by adding different amounts of doxycycline to the culture (Fig 2). The * reaction closes the loop and is used to model the circuit that has positive feedback. The ** reaction is not used for the models with positive feedback. The fifth reaction defines Tat’s modulation of promoter toggling. For Tat affecting k_OFF_, Tat binds to the LTR_ON_ state and creates a third state, LTR_TatON_. From the LTR_TatON_ state, the LTR must move through LTR_ON_ first before switching back OFF.
(PDF)

S2 Table Chemical reaction scheme (with parameters) for stochastic simulations of circuits where transactivator of transcription (Tat) only modulates k_ON_. See the S1 Table description for further information. The fifth reaction represents Tat’s ability to modulate burst frequency through k_ON_. Tat binds to the LTR_OFF_ state and flips the promoter to the LTR_ON_ state.
(PDF)

S3 Table Chemical reaction scheme (with parameters) for stochastic simulations of circuits where transactivator of transcription (Tat) only modulates alpha. See the S1 Table description for further information. The fifth reaction defines Tat’s modulation of burst size by modulating alpha. Tat binds to the LTR_ON_ state and promotes transcription, thereby affecting transcriptional efficiency when the promoter is already in an active state.
(PDF)

S1 Text The effect of feedback on bimodality robustness. The supplementary text provides the derivation and assumptions behind Eq 1 and Eq 2 and Fig 5 of the main text.
(DOCX)

## Abbreviations

CTD: carboxy-terminal domain
eGFP: enhanced green fluorescent protein
FB: feedback strength
GFP: green fluorescent protein
HDACi: histone deacetylase inhibitor
LRA: latency-reversing agent
LTR: long terminal repeat
NFkB: nuclear factor kappa-light-chain-enhancer of activated B cells
PKC: protein kinase C
pTEFb: positive transcriptional elongation factor b
RNAPII: RNA polymerase II
TAR: Tat-activation RNA
TF: transcription factor
TNFα: tumor necrosis factor alpha
Tat: transactivator of transcription

## References

[1] Balázsi G, van Oudenaarden A, Collins JJ. Cellular decision making and biological noise: from microbes to mammals. CELL. 2011;144: 910–925. doi: 10.1016/j.cell.2011.01.030 21414483 10.1016/j.cell.2011.01.030 PMC3068611

[2] Blake WJ, Kærn M, Cantor CR, Collins JJ. Noise in eukaryotic gene expression. Nature. Nature Publishing Group; 2003;422: 633–637. doi: 10.1038/nature01546 12687005 10.1038/nature01546

[3] Shalek AK, Satija R, Adiconis X, Gertner RS, Gaublomme JT, Raychowdhury R, et al Single-cell transcriptomics reveals bimodality in expression and splicing in immune cells. Nature. Nature Research; 2013;498: 236–240. doi: 10.1038/nature12172 23685454 10.1038/nature12172 PMC3683364

[4] Ochab-Marcinek A, Tabaka M. Bimodal gene expression in noncooperative regulatory systems. Proc Natl Acad Sci USA. National Acad Sciences; 2010;107: 22096–22101. doi: 10.1073/pnas.1008965107 21135209 10.1073/pnas.1008965107 PMC3009792

[5] Raj A, Rifkin SA, Andersen E, van Oudenaarden A. Variability in gene expression underlies incomplete penetrance. Nature. Nature Publishing Group; 2010;463: 913–918. doi: 10.1038/nature08781 20164922 10.1038/nature08781 PMC2836165

[6] Isaacs FJ, Hasty J, Cantor CR, Collins JJ. Prediction and measurement of an autoregulatory genetic module. Proc Natl Acad Sci USA. National Acad Sciences; 2003;100: 7714–7719. doi: 10.1073/pnas.1332628100 12808135 10.1073/pnas.1332628100 PMC164653

[7] Lipshtat A, Loinger A, Balaban NQ, Biham O. Genetic Toggle Switch without Cooperative Binding. Phys Rev Lett. American Physical Society; 2006;96: 188101 doi: 10.1103/PhysRevLett.96.188101 16712399 10.1103/PhysRevLett.96.188101

[8] SUel GM, Garcia-Ojalvo J, Liberman LM, Elowitz MB. An excitable gene regulatory circuit induces transient cellular differentiation. Nature. 2006;440: 545–550. doi: 10.1038/nature04588 16554821 10.1038/nature04588

[9] Barkai N, Leibler S. Robustness in simple biochemical networks. Nature. Nature Publishing Group; 1997;387: 913–917. doi: 10.1038/43199 9202124 10.1038/43199

[10] Weinberger LS. A minimal fate-selection switch. Current Opinion in Cell Biology. 2015;37: 111–118. doi: 10.1016/j.ceb.2015.10.005 26611210 10.1016/j.ceb.2015.10.005

[11] Hartwell LH, Hopfield JJ, Leibler S, Murray AW. From molecular to modular cell biology. Nature. Nature Publishing Group; 1999;402: C47–52. doi: 10.1038/35011540 10591225 10.1038/35011540

[12] Ferrell JE Jr. Self-perpetuating states in signal transduction: positive feedback, double-negative feedback and bistability. Current Opinion in Cell Biology. 2002;14: 140–148. doi: 10.1016/S0955-0674(02)00314-9 11891111 10.1016/s0955-0674(02)00314-9

[13] Ferrell JE Jr, Ha SH. Ultrasensitivity part I: Michaelian responses and zero-order ultrasensitivity. Trends in Biochemical Sciences. 2014;39: 496–503. doi: 10.1016/j.tibs.2014.08.003 25240485 10.1016/j.tibs.2014.08.003 PMC4214216

[14] Ferrell JE Jr, Ha SH. Ultrasensitivity part II: multisite phosphorylation, stoichiometric inhibitors, and positive feedback. Trends in Biochemical Sciences. 2014;39: 556–569. doi: 10.1016/j.tibs.2014.09.003 25440716 10.1016/j.tibs.2014.09.003 PMC4435807

[15] Ferrell JE Jr, Ha SH. Ultrasensitivity part III: cascades, bistable switches, and oscillators. Trends in Biochemical Sciences. Elsevier Ltd; 2014;: 1–7. doi: 10.1016/j.tibs.2014.10.002 25456048 10.1016/j.tibs.2014.10.002 PMC4254632

[16] Brandman O, Meyer T. Feedback loops shape cellular signals in space and time. Science. American Association for the Advancement of Science; 2008;322: 390–395. doi: 10.1126/science.1160617 18927383 10.1126/science.1160617 PMC2680159

[17] Ferrell JE Jr. Self-perpetuating states in signal transduction: positive feedback, double-negative feedback and bistability. Current Opinion in Cell Biology. 2002;14: 140–148. doi: 10.1016/S0955-0674(02)00314-9 11891111 10.1016/s0955-0674(02)00314-9

[18] Sanchez A, Golding I. Genetic Determinants and Cellular Constraints in Noisy Gene Expression. Science. 2013;342: 1188–1193. doi: 10.1126/science.1242975 24311680 10.1126/science.1242975 PMC4045091

[19] Shahrezaei V, Swain PS. Analytical distributions for stochastic gene expression. Proc Natl Acad Sci USA. National Acad Sciences; 2008;105: 17256–17261. doi: 10.1073/pnas.0803850105 18988743 10.1073/pnas.0803850105 PMC2582303

[20] Kærn M, Elston TC, Blake WJ, Collins JJ. Stochasticity in gene expression: from theories to phenotypes. Nat Rev Genet. 2005;6: 451–464. doi: 10.1038/nrg1615 15883588 10.1038/nrg1615

[21] Xu H, Skinner SO, Sokac AM, Golding I. Stochastic Kinetics of Nascent RNA. Phys Rev Lett. 2016;117: 128101 doi: 10.1103/PhysRevLett.117.128101 27667861 10.1103/PhysRevLett.117.128101 PMC5033037

[22] Senecal A, Munsky B, Proux F, Ly N, Braye FE, Zimmer C, et al Transcription factors modulate c-Fos transcriptional bursts. CellReports. 2014;8: 75–83. doi: 10.1016/j.celrep.2014.05.053 24981864 10.1016/j.celrep.2014.05.053 PMC5555219

[23] Xu H, Sepúlveda LA, Figard L, Sokac AM, Golding I. Combining protein and mRNA quantification to decipher transcriptional regulation. Nature Publishing Group. Nature Research; 2015;12: 739–742. doi: 10.1038/nmeth.3446 26098021 10.1038/nmeth.3446 PMC4521975

[24] Dar RD, Razooky BS, Singh A, Trimeloni TV, McCollum JM, Cox CD, et al Transcriptional burst frequency and burst size are equally modulated across the human genome. Proc Natl Acad Sci USA. National Acad Sciences; 2012;109: 17454–17459. doi: 10.1073/pnas.1213530109 23064634 10.1073/pnas.1213530109 PMC3491463

[25] Suter DM, Molina N, Gatfield D, Schneider K, Schibler U, Naef F. Mammalian Genes Are Transcribed with Widely Different Bursting Kinetics. Science. 2011;332: 472–474. doi: 10.1126/science.1198817 21415320 10.1126/science.1198817

[26] To T-L, Maheshri N. Noise can induce bimodality in positive transcriptional feedback loops without bistability. Science. American Association for the Advancement of Science; 2010;327: 1142–1145. doi: 10.1126/science.1178962 20185727 10.1126/science.1178962

[27] Razooky BS, Pai A, Aull K, Rouzine IM, Weinberger LS. A hardwired HIV latency program. CELL. 2015;160: 990–1001. doi: 10.1016/j.cell.2015.02.009 25723172 10.1016/j.cell.2015.02.009 PMC4395878

[28] Finzi D, Hermankova M, Pierson T, Carruth LM, Buck C, Chaisson RE, et al Identification of a reservoir for HIV-1 in patients on highly active antiretroviral therapy. Science. 1997;278: 1295–1300. 9360927 10.1126/science.278.5341.1295

[29] Siliciano RF, Greene WC. HIV Latency. Cold Spring Harbor Perspectives in Medicine. 2011;1: a007096–a007096. doi: 10.1101/cshperspect.a007096 22229121 10.1101/cshperspect.a007096 PMC3234450

[30] Kao SY, Calman AF, Luciw PA, Peterlin BM. Anti-termination of transcription within the long terminal repeat of HIV-1 by tat gene product. Nature. Nature Publishing Group; 1987;330: 489–493. doi: 10.1038/330489a0 2825027 10.1038/330489a0

[31] Mancebo HSY, Lee G, Flygare J, Tomassini J, Luu P, Zhu Y, et al P-TEFb kinase is required for HIV Tat transcriptional activation in vivo and in vitro. Genes & Development. 1997;11: 2633–2644. doi: 10.1101/gad.11.20.2633 9334326 10.1101/gad.11.20.2633 PMC316604

[32] Weinberger LS, Burnett JC, Toettcher JE, Arkin AP, Schaffer DV. Stochastic Gene Expression in a Lentiviral Positive-Feedback Loop: HIV-1 Tat Fluctuations Drive Phenotypic Diversity. CELL. 2005;122: 169–182. doi: 10.1016/j.cell.2005.06.006 16051143 10.1016/j.cell.2005.06.006

[33] Ozbudak EM, Thattai M, Kurtser I, Grossman AD, van Oudenaarden A. Regulation of noise in the expression of a single gene. Nat Genet. 2002;31: 69–73. doi: 10.1038/ng869 11967532 10.1038/ng869

[34] Ferrell JE, Xiong W. Bistability in cell signaling: How to make continuous processes discontinuous, and reversible processes irreversible. Chaos. 2001;11: 227–10. doi: 10.1063/1.1349894 12779456 10.1063/1.1349894

[35] Weinberger LS, Shenk T. An HIV Feedback Resistor: Auto-Regulatory Circuit Deactivator and Noise Buffer. Aitchison JD, editor. PLoS Biol. 2006;5: e9–15. doi: 10.1371/journal.pbio.0050009 17194214 10.1371/journal.pbio.0050009 PMC1717016

[36] Singh A, Razooky B, Cox CD, Simpson ML, Weinberger LS. Transcriptional bursting from the HIV-1 promoter is a significant source of stochastic noise in HIV-1 gene expression. Biophys J. 2010;98: L32–4. doi: 10.1016/j.bpj.2010.03.001 20409455 10.1016/j.bpj.2010.03.001 PMC2856162

[37] Skupsky R, Burnett JC, Foley JE, Schaffer DV, Arkin AP. HIV promoter integration site primarily modulates transcriptional burst size rather than frequency. Friedman N, editor. PLoS Comput Biol. 2010;6: e1000952 doi: 10.1371/journal.pcbi.1000952 20941390 10.1371/journal.pcbi.1000952 PMC2947985

[38] Rouzine IM, Weinberger AD, Weinberger LS. An evolutionary role for HIV latency in enhancing viral transmission. CELL. 2015;160: 1002–1012. doi: 10.1016/j.cell.2015.02.017 25723173 10.1016/j.cell.2015.02.017 PMC4488136

[39] Hartigan JA, Hartigan PM. The Dip Test of Unimodality on JSTOR. The Annals of Statistics. 1985 doi: 10.2307/2241144

[40] Weinberger LS, Dar RD, Simpson ML. Transient-mediated fate determination in a transcriptional circuit of HIV. Nat Genet. 2008;40: 466–470. doi: 10.1038/ng.116 18344999 10.1038/ng.116

[41] Siliciano RF, Greene WC. HIV Latency. Cold Spring Harbor Perspectives in Medicine. 2011;1: a007096–a007096. doi: 10.1101/cshperspect.a007096 22229121 10.1101/cshperspect.a007096 PMC3234450

[42] Jordan A, Defechereux P, Verdin E. The site of HIV-1 integration in the human genome determines basal transcriptional activity and response to Tat transactivation. EMBO J. EMBO Press; 2001;20: 1726–1738. doi: 10.1093/emboj/20.7.1726 11285236 10.1093/emboj/20.7.1726 PMC145503

[43] Birtwistle MR, Kriegsheim von A, Dobrzyński M, Kholodenko BN, Kolch W. Mammalian protein expression noise: scaling principles and the implications for knockdown experiments. Mol BioSyst. Royal Society of Chemistry; 2012;8: 3068–3076. doi: 10.1039/C2MB25168J 22990612 10.1039/c2mb25168j

[44] Simpson ML, Cox CD, Sayler GS. Frequency domain analysis of noise in autoregulated gene circuits. Proc Natl Acad Sci USA. 2003;100: 4551–4556. doi: 10.1073/pnas.0736140100 12671069 10.1073/pnas.0736140100 PMC404696

[45] Austin DW, Allen MS, McCollum JM, Dar RD, Wilgus JR, Sayler GS, et al Gene network shaping of inherent noise spectra. Nature. Nature Publishing Group; 2006;439: 608–611. doi: 10.1038/nature04194 16452980 10.1038/nature04194

[46] Soboleski MR, Oaks J, Halford WP. Green fluorescent protein is a quantitative reporter of gene expression in individual eukaryotic cells. FASEB J. Federation of American Societies for Experimental Biology; 2005;19: 440–442. doi: 10.1096/fj.04-3180fje 15640280 10.1096/fj.04-3180fje PMC1242169

[47] Kitano H. Biological robustness. Nat Rev Genet. 2004;5: 826–837. doi: 10.1038/nrg1471 15520792 10.1038/nrg1471

[48] Weinberger AD, Weinberger LS. Stochastic fate selection in HIV-infected patients. CELL. 2013;155: 497–499. doi: 10.1016/j.cell.2013.09.039 24243007 10.1016/j.cell.2013.09.039

[49] Ho Y-C, Shan L, Hosmane NN, Wang J, Laskey SB, Rosenbloom DIS, et al Replication-competent noninduced proviruses in the latent reservoir increase barrier to HIV-1 cure. CELL. 2013;155: 540–551. doi: 10.1016/j.cell.2013.09.020 24243014 10.1016/j.cell.2013.09.020 PMC3896327

[50] Bar-Even A, Paulsson J, Maheshri N, Carmi M, O'Shea E, Pilpel Y, et al Noise in protein expression scales with natural protein abundance. Nat Genet. 2006;38: 636–643. doi: 10.1038/ng1807 16715097 10.1038/ng1807

[51] Miyashiro T, Goulian M. High stimulus unmasks positive feedback in an autoregulated bacterial signaling circuit. Proc Natl Acad Sci USA. National Acad Sciences; 2008;105: 17457–17462. doi: 10.1073/pnas.0807278105 18987315 10.1073/pnas.0807278105 PMC2582279

[52] Tsai TY-C, Choi YS, Ma W, Pomerening JR, Tang C, Ferrell JE. Robust, tunable biological oscillations from interlinked positive and negative feedback loops. Science. American Association for the Advancement of Science; 2008;321: 126–129. doi: 10.1126/science.1156951 18599789 10.1126/science.1156951 PMC2728800

[53] Lim WA, Lee CM, Tang C. Design principles of regulatory networks: searching for the molecular algorithms of the cell. Molecular Cell. 2013;49: 202–212. doi: 10.1016/j.molcel.2012.12.020 23352241 10.1016/j.molcel.2012.12.020 PMC3664230

[54] Dar RD, Hosmane NN, Arkin MR, Siliciano RF, Weinberger LS. Screening for noise in gene expression identifies drug synergies. Science. American Association for the Advancement of Science; 2014;344: 1392–1396. doi: 10.1126/science.1250220 24903562 10.1126/science.1250220 PMC4122234

[55] Dull T, Zufferey R, Kelly M, Mandel RJ, Nguyen M, Trono D, et al A third-generation lentivirus vector with a conditional packaging system. Journal of Virology. American Society for Microbiology (ASM); 1998;72: 8463–8471. 10.1128/jvi.72.11.8463-8471.1998 PMC110254 9765382

[56] Gillespie DT. Exact stochastic simulation of coupled chemical reactions. The journal of physical chemistry. 1977.

[57] Cao Y, Terebus A, Liang J. Accurate Chemical Master Equation Solution Using Multi-Finite Buffers. Multiscale Model Simul. 2016;14: 923–963. doi: 10.1137/15M1034180 27761104 10.1137/15M1034180 PMC5066912

[58] Cao Y, Terebus A, Liang J. State Space Truncation with Quantified Errors for Accurate Solutions to Discrete Chemical Master Equation. Bull Math Biol. 3rd ed. Springer US; 2016;78: 617–661. doi: 10.1007/s11538-016-0149-1 27105653 10.1007/s11538-016-0149-1 PMC4896403

[59] Wand MP, Jones MC. Kernel smoothing. 1994.

