# Supplementary figures and images for: Nonlatching positive feedback enables robust bimodality by decoupling expression noise from the mean
Source: PLoS Biol. 2017 Oct 18;15(10):e2000841. doi: 10.1371/journal.pbio.2000841 (PMC5646755; doi:10.1371/journal.pbio.2000841)

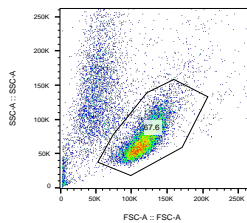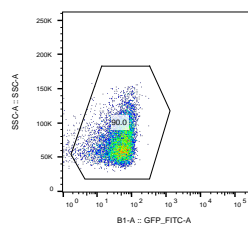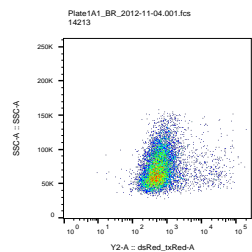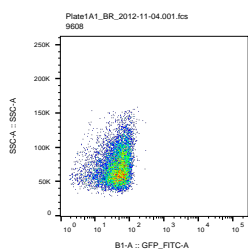

Plate1A1\_BR\_2012-11-04.001.fcs  
8650

Plate1A1\_BR\_2012-11-04.001.fcs  
8650

Supplement: S21 Data — The forward-scatter and side-scatter values were used to determine the live population (top left). The live population was gated on green fluorescent protein (GFP) values over the axis (top right) to then quantify the mCherry values (bottom left) and GFP values (bottom right). The gating strategy in S21 Data applied to the flow cytometry files (S10 Data) and was used to generate the numbers in Fig 2B and S2, S3, S12 and S13 Figs. (PDF) [file pbio.2000841.s021.pdf]

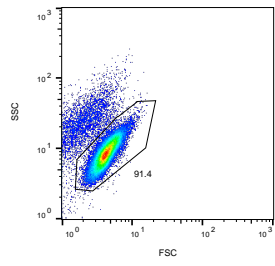

3 iso .5x.FCS  
55543

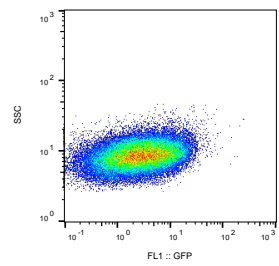

3 iso .5x.FCS  
50784

Supplement: S23 Data — The forward-scatter and side-scatter values were used to determine the live population (left). The live population green fluorescent protein (GFP) values were used to generate the data in S8 and S10 Figs. (PDF) [file pbio.2000841.s023.pdf]

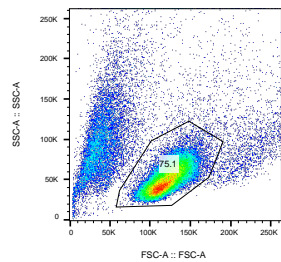

Plate1F2\_BR\_2012-10-13.001.fcs  
54219

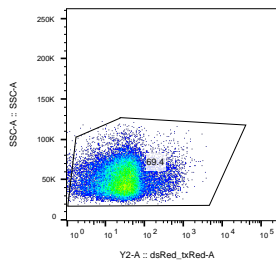

Plate1F2\_BR\_2012-10-13.001.fcs  
40745

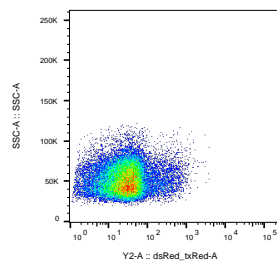

Plate1F2\_BR\_2012-10-13.001.fcs  
28290

Supplement: S28 Data — The forward-scatter and side-scatter values were used to determine the live population (left). The live mCherry population was then gated to remove debris that fluoresced at the axis (middle graph), and the mCherry values were extracted. These values were used in Fig 3B and 3C and S7 and S11 Figs and can be found in S29 Data. (PDF) [file pbio.2000841.s028.pdf]

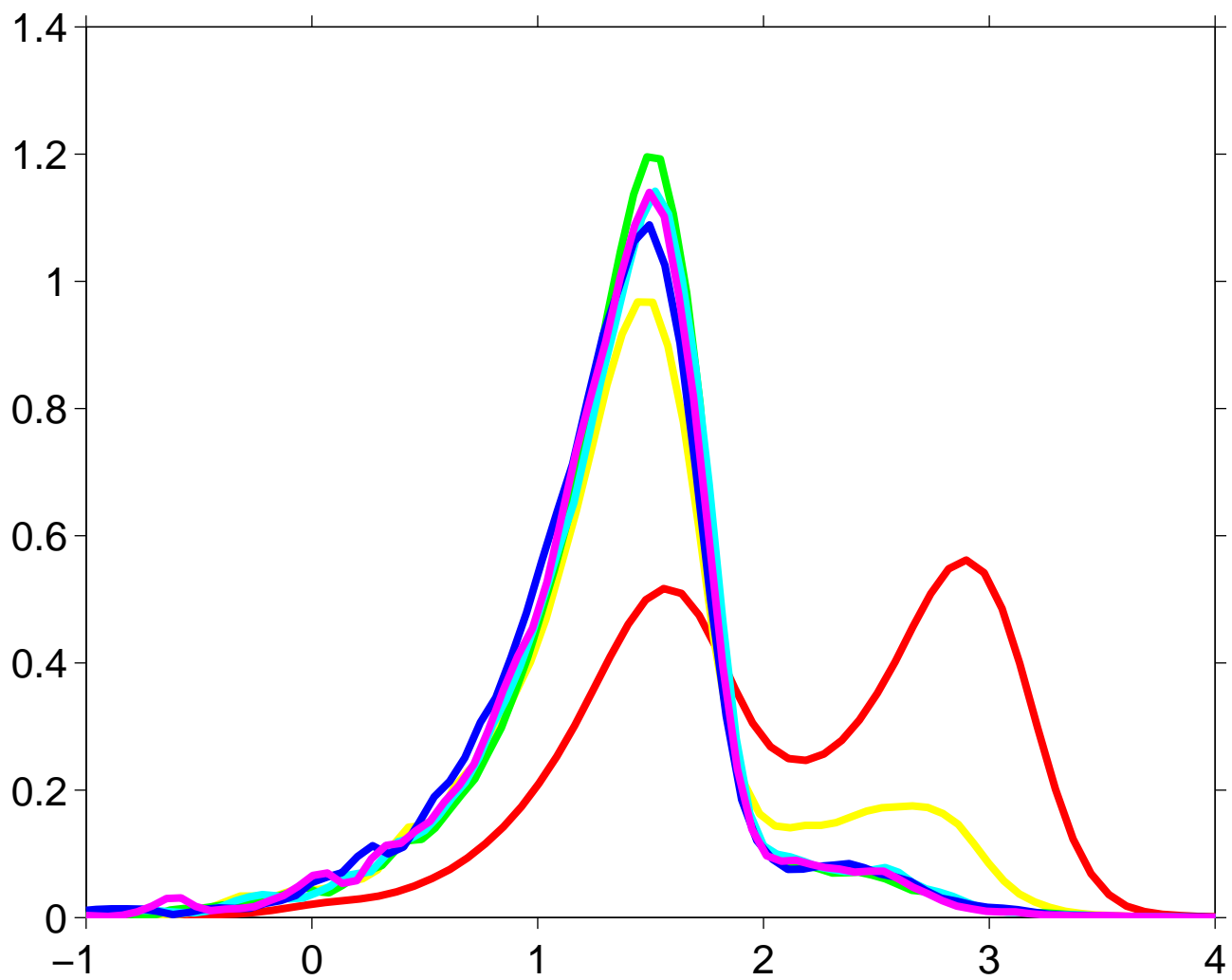

Supplement: S29 Data — The corresponding mCherry fluorescence values were taken according to the gating strategy in S28 Data applied to the flow cytometry files (S27 Data) and were used to generate the numbers in Fig 3B and 3C and S7 and S11 Figs. (ZIP) [file pbio.2000841.s029.zip › S29_Data/LChITF Iso 1/overpdfplotR1toR6.pdf]

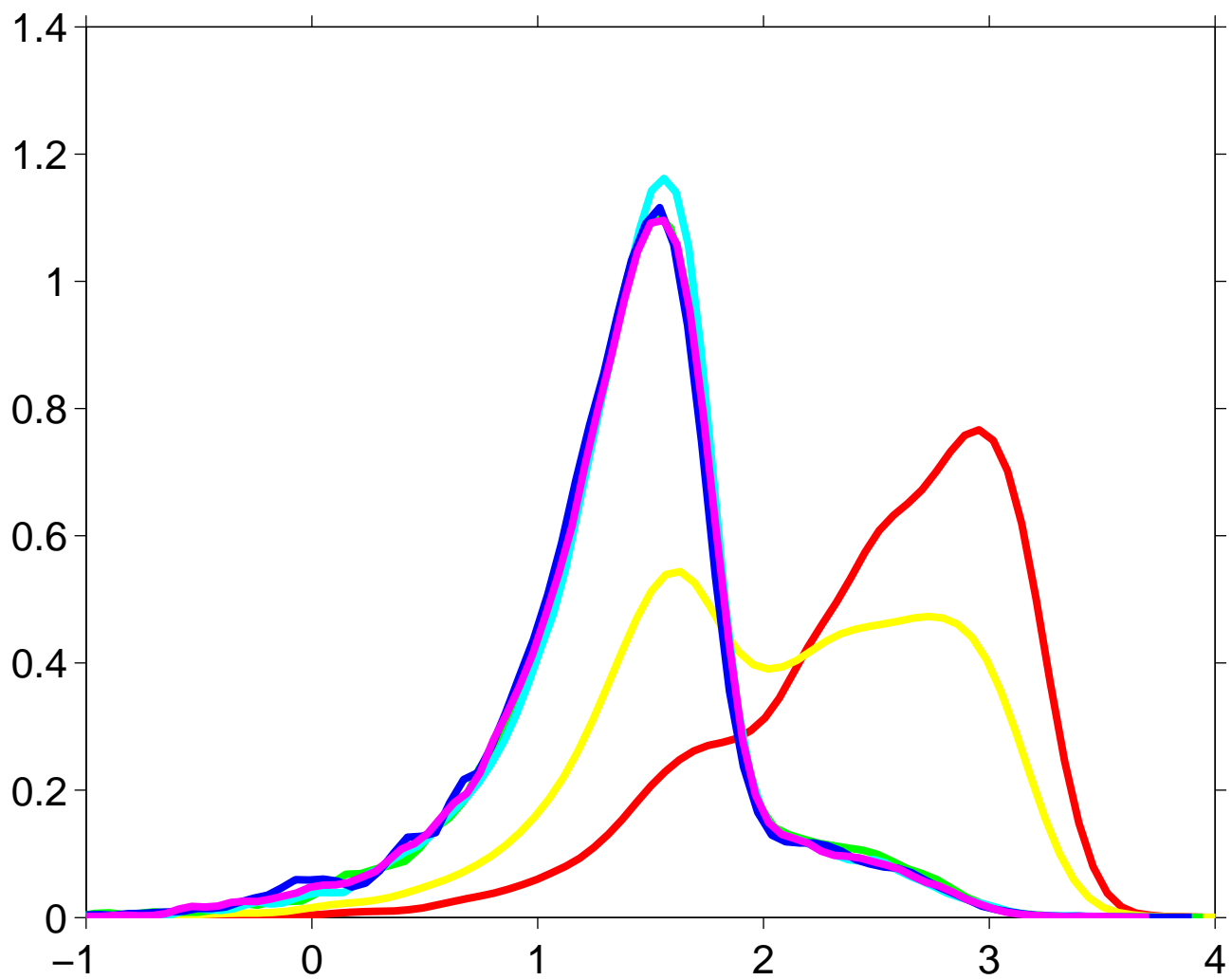

Supplement: S29 Data — The corresponding mCherry fluorescence values were taken according to the gating strategy in S28 Data applied to the flow cytometry files (S27 Data) and were used to generate the numbers in Fig 3B and 3C and S7 and S11 Figs. (ZIP) [file pbio.2000841.s029.zip › S29_Data/LChITF Iso 2/overpdfplotR1toR6.pdf]

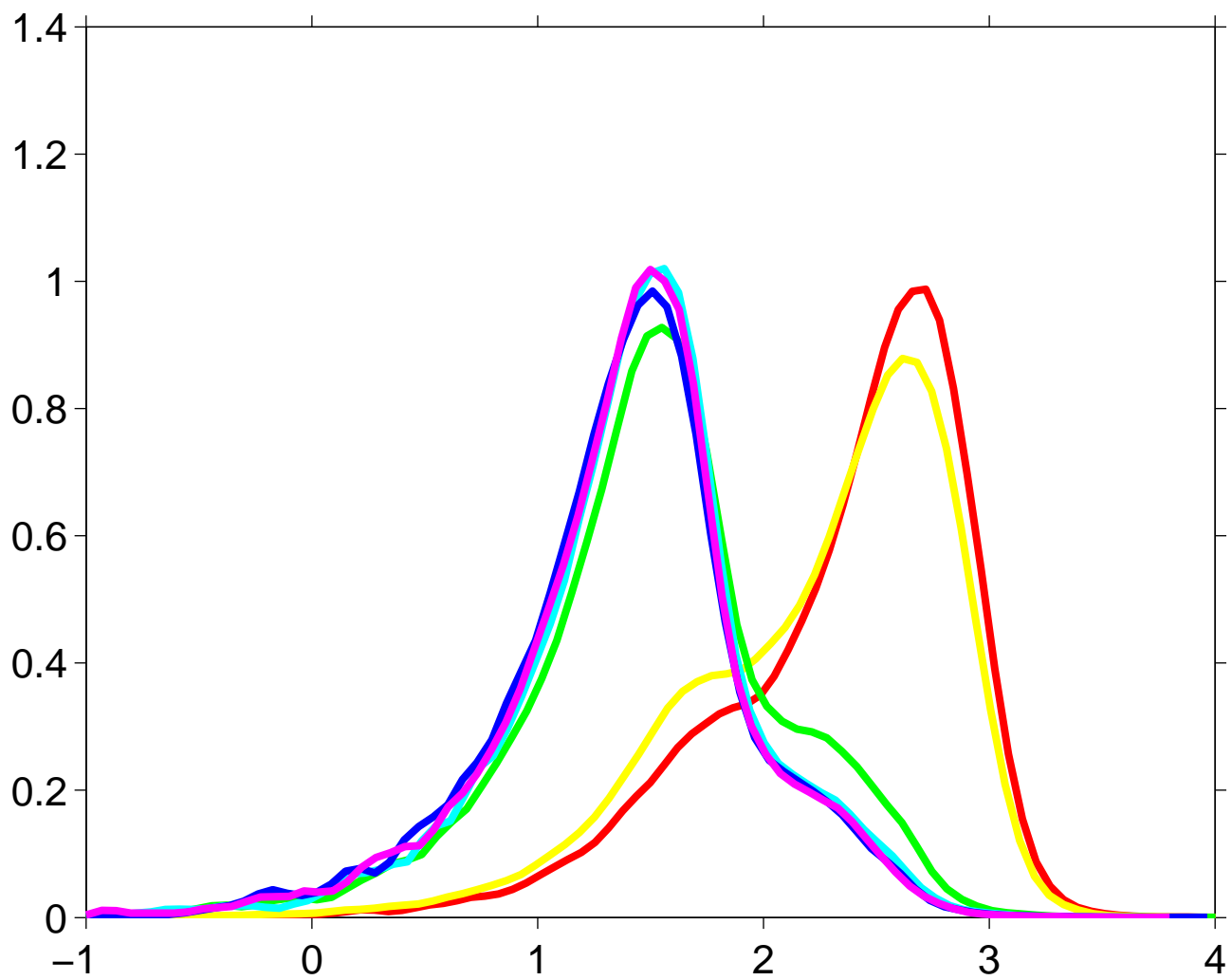

Supplement: S29 Data — The corresponding mCherry fluorescence values were taken according to the gating strategy in S28 Data applied to the flow cytometry files (S27 Data) and were used to generate the numbers in Fig 3B and 3C and S7 and S11 Figs. (ZIP) [file pbio.2000841.s029.zip › S29_Data/LChITF Iso 3/overpdfplotR1toR6.pdf]

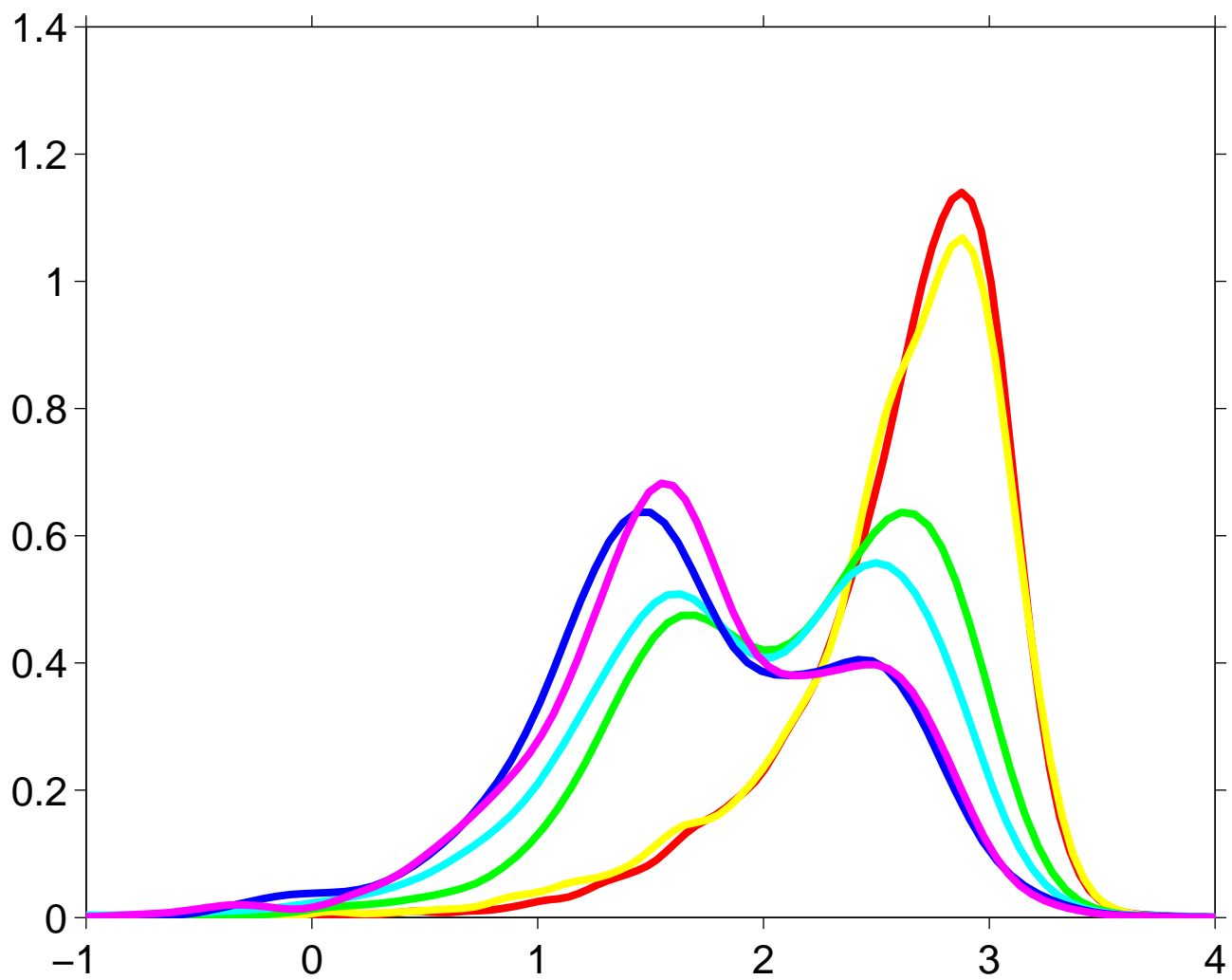

Supplement: S29 Data — The corresponding mCherry fluorescence values were taken according to the gating strategy in S28 Data applied to the flow cytometry files (S27 Data) and were used to generate the numbers in Fig 3B and 3C and S7 and S11 Figs. (ZIP) [file pbio.2000841.s029.zip › S29_Data/LChITF Iso 4/overpdfplotR1toR6.pdf]

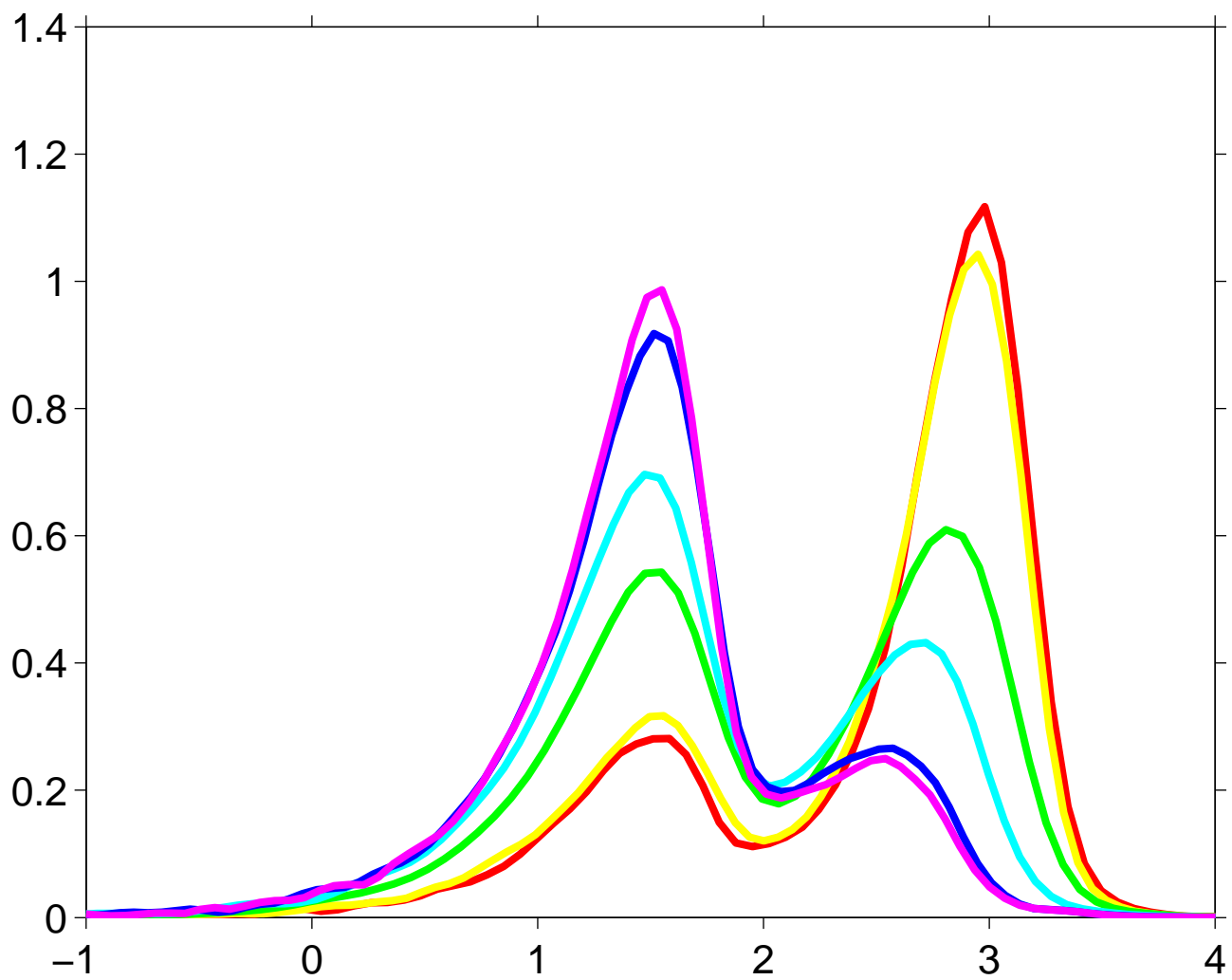

Supplement: S29 Data — The corresponding mCherry fluorescence values were taken according to the gating strategy in S28 Data applied to the flow cytometry files (S27 Data) and were used to generate the numbers in Fig 3B and 3C and S7 and S11 Figs. (ZIP) [file pbio.2000841.s029.zip › S29_Data/LChITF Iso 5/overpdfplotR1toR6.pdf]

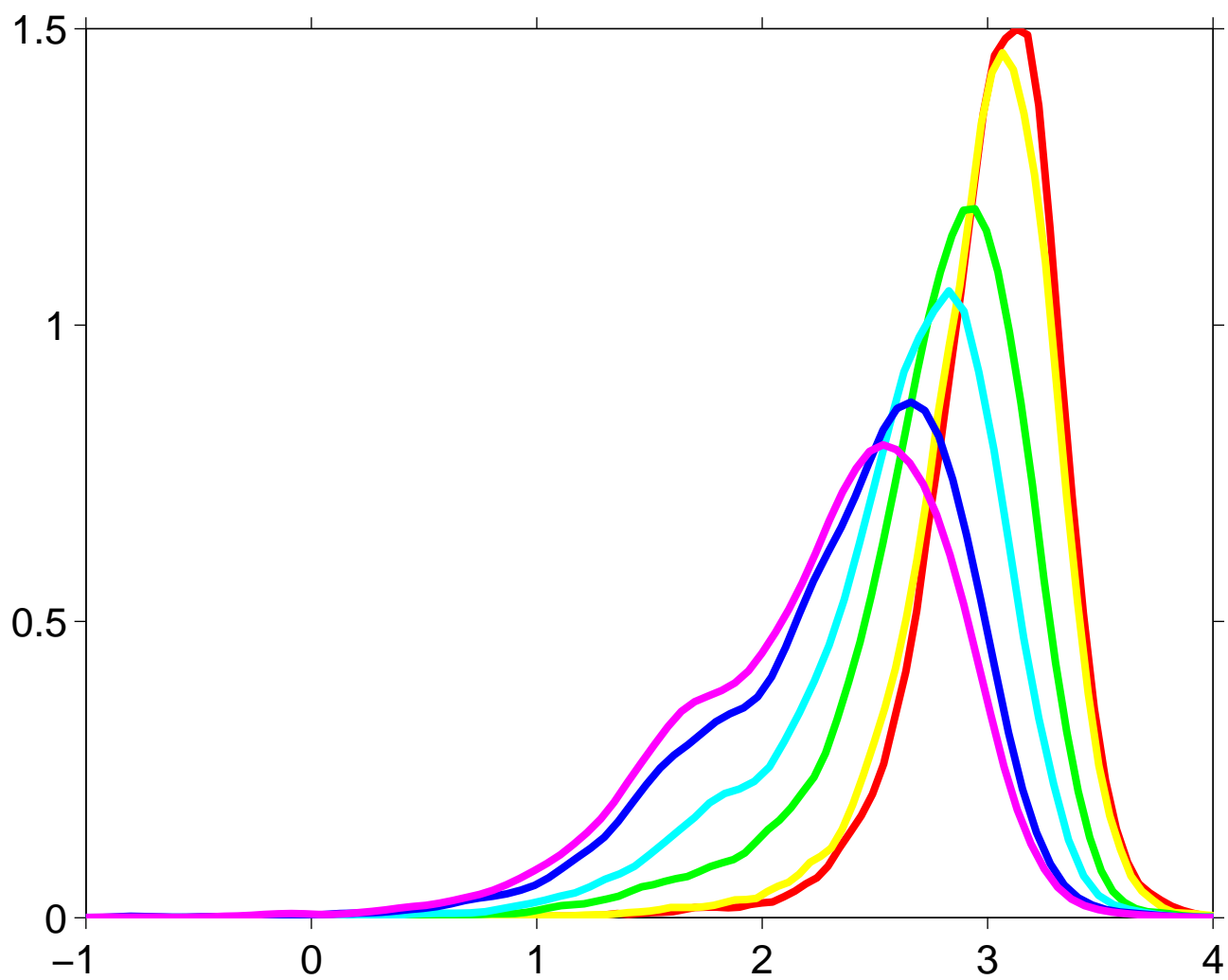

Supplement: S29 Data — The corresponding mCherry fluorescence values were taken according to the gating strategy in S28 Data applied to the flow cytometry files (S27 Data) and were used to generate the numbers in Fig 3B and 3C and S7 and S11 Figs. (ZIP) [file pbio.2000841.s029.zip › S29_Data/LChITF Iso 6/overpdfplotR1toR6.pdf]

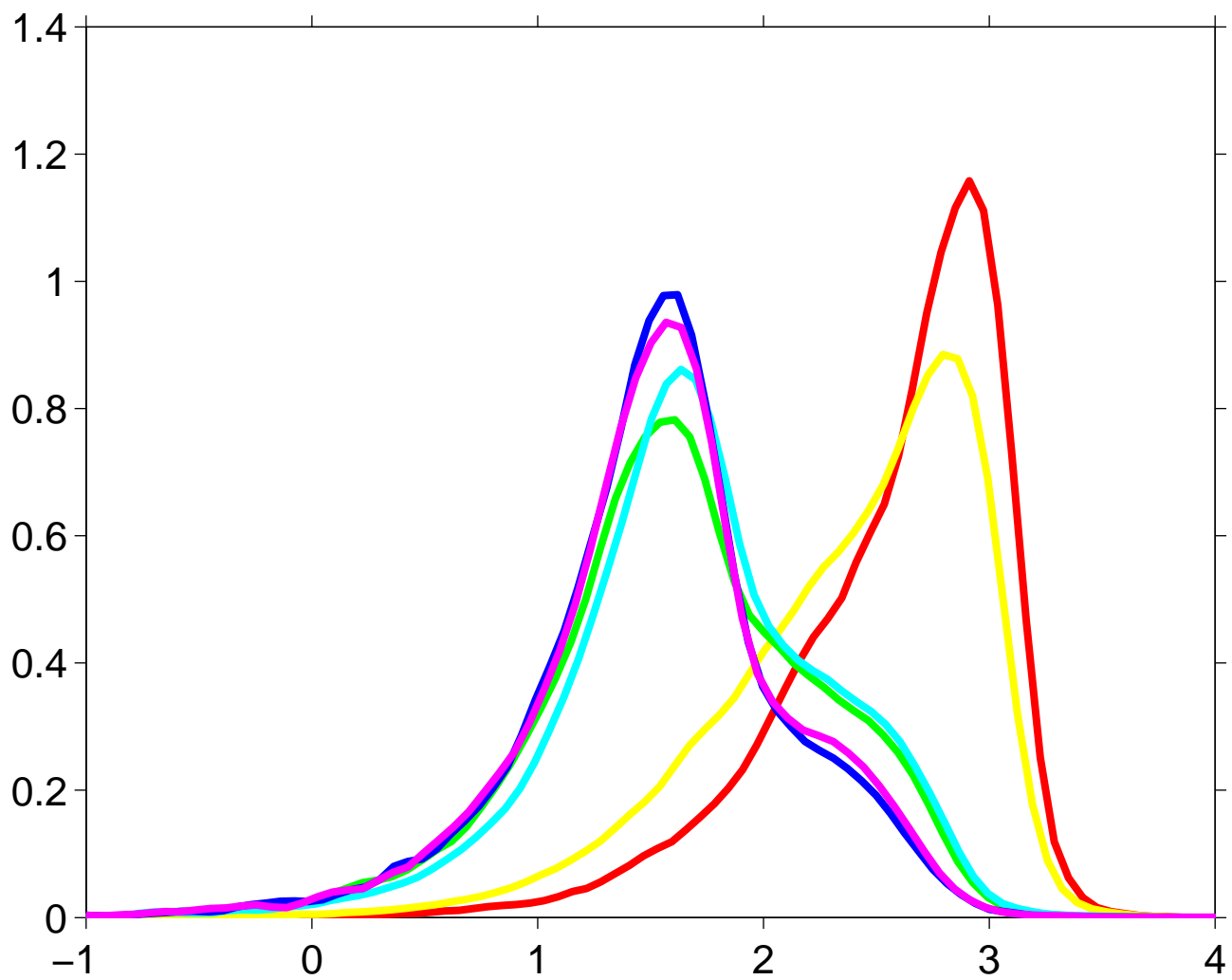

Supplement: S29 Data — The corresponding mCherry fluorescence values were taken according to the gating strategy in S28 Data applied to the flow cytometry files (S27 Data) and were used to generate the numbers in Fig 3B and 3C and S7 and S11 Figs. (ZIP) [file pbio.2000841.s029.zip › S29_Data/LChITF Iso 7/overpdfplotR1toR6.pdf]

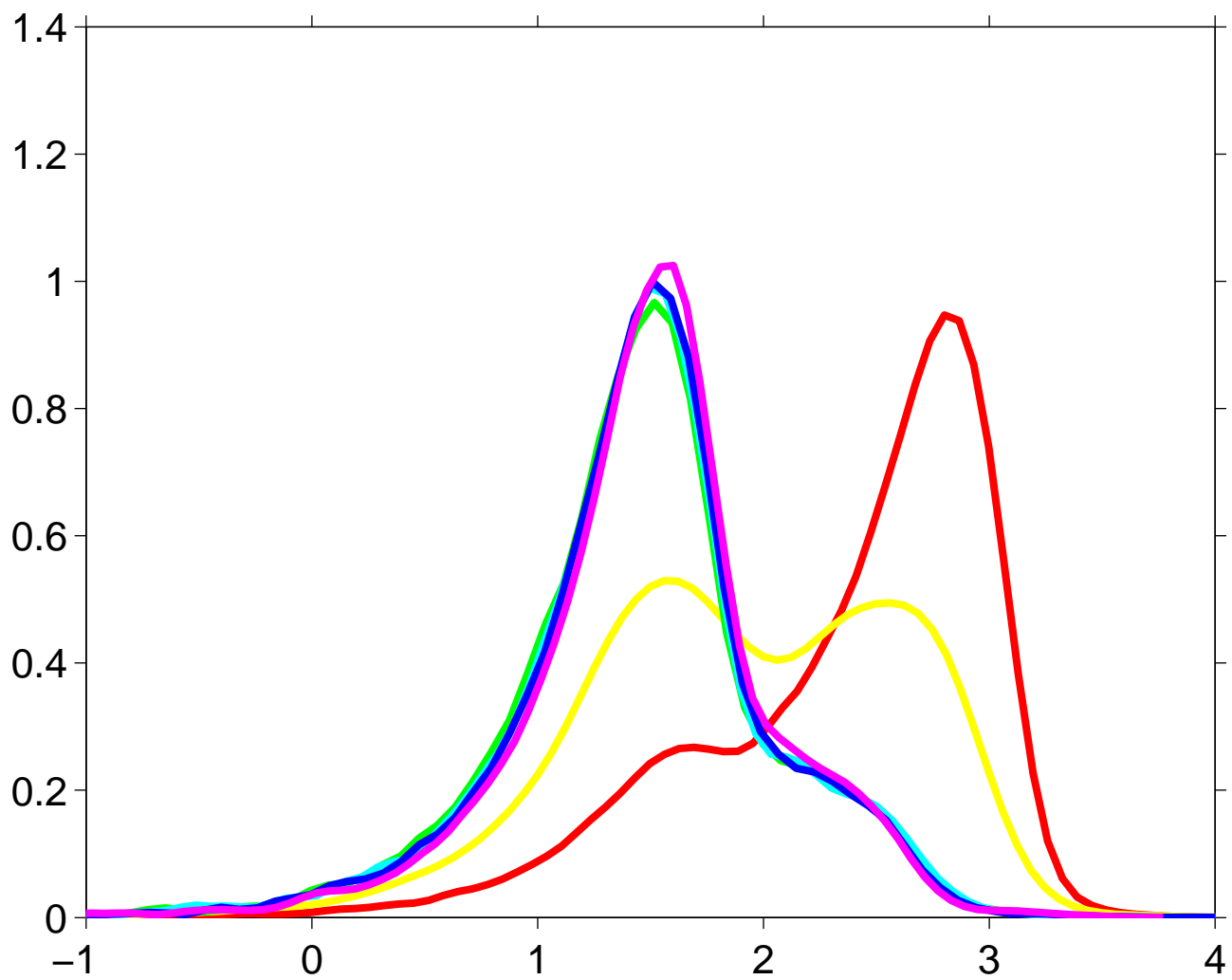

Supplement: S29 Data — The corresponding mCherry fluorescence values were taken according to the gating strategy in S28 Data applied to the flow cytometry files (S27 Data) and were used to generate the numbers in Fig 3B and 3C and S7 and S11 Figs. (ZIP) [file pbio.2000841.s029.zip › S29_Data/LChITF Iso 8/overpdfplotR1toR6.pdf]

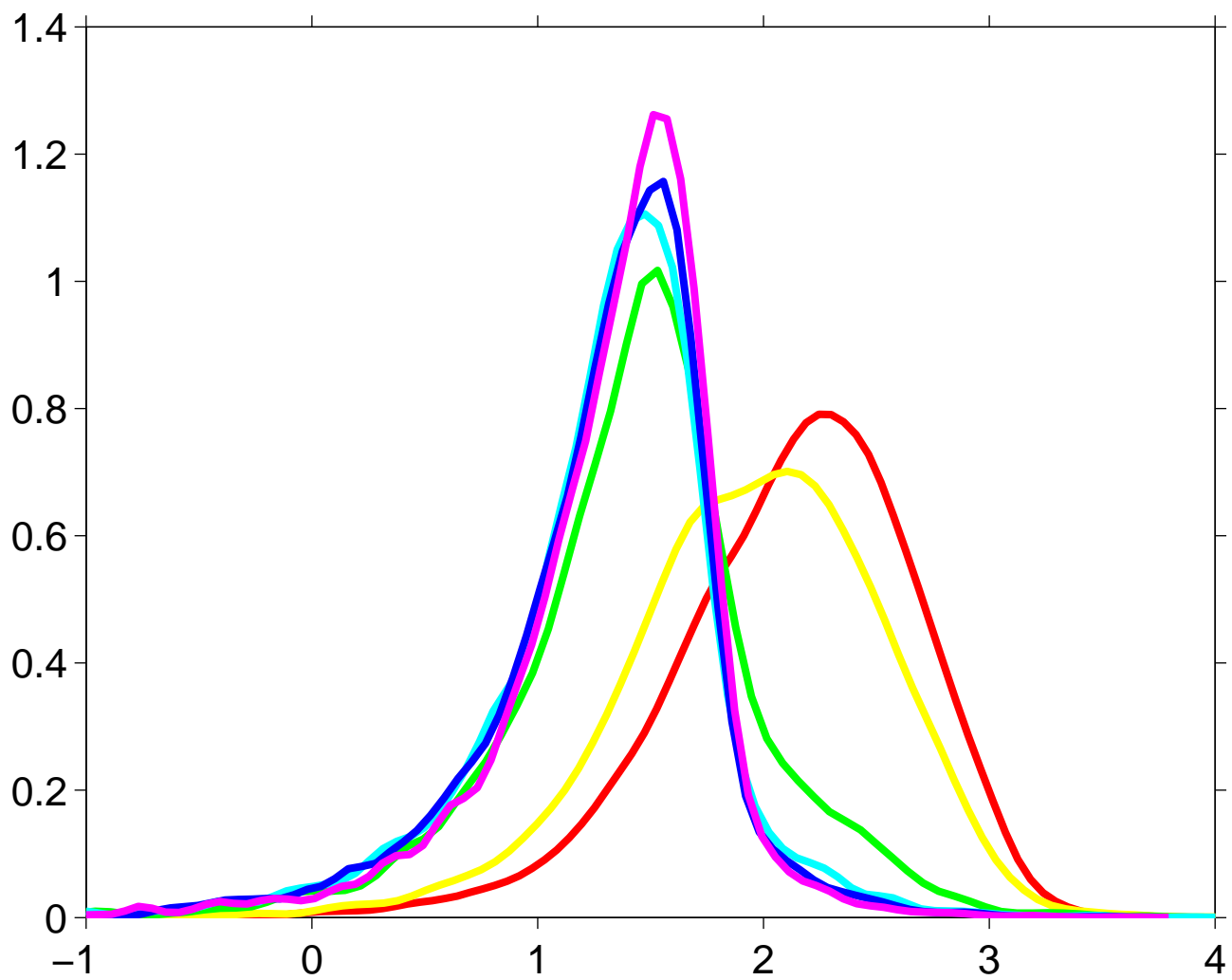

Supplement: S29 Data — The corresponding mCherry fluorescence values were taken according to the gating strategy in S28 Data applied to the flow cytometry files (S27 Data) and were used to generate the numbers in Fig 3B and 3C and S7 and S11 Figs. (ZIP) [file pbio.2000841.s029.zip › S29_Data/LChITF Iso 9/overpdfplotR1toR6.pdf]

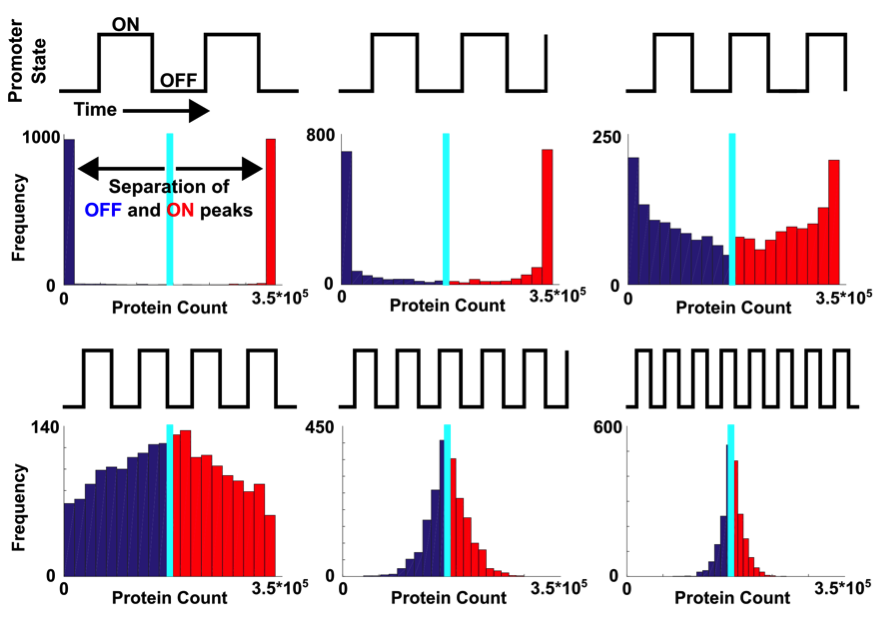

Supplement: S1 Fig — For a given time, the rate of switching between the ON and OFF promoter states (top pulse trains) is related to the duration of time in a specific promoter state. The duration of the promoter state determines the length, or separation from the mean (cyan line, same value for each panel), of the transient production or decay of gene-expression products. Increasing promoter kinetics reduces transients and the separation between potential peaks in a bursty system (top left moving to the right and then bottom left moving to the right). (TIF) [file pbio.2000841.s036.tif]

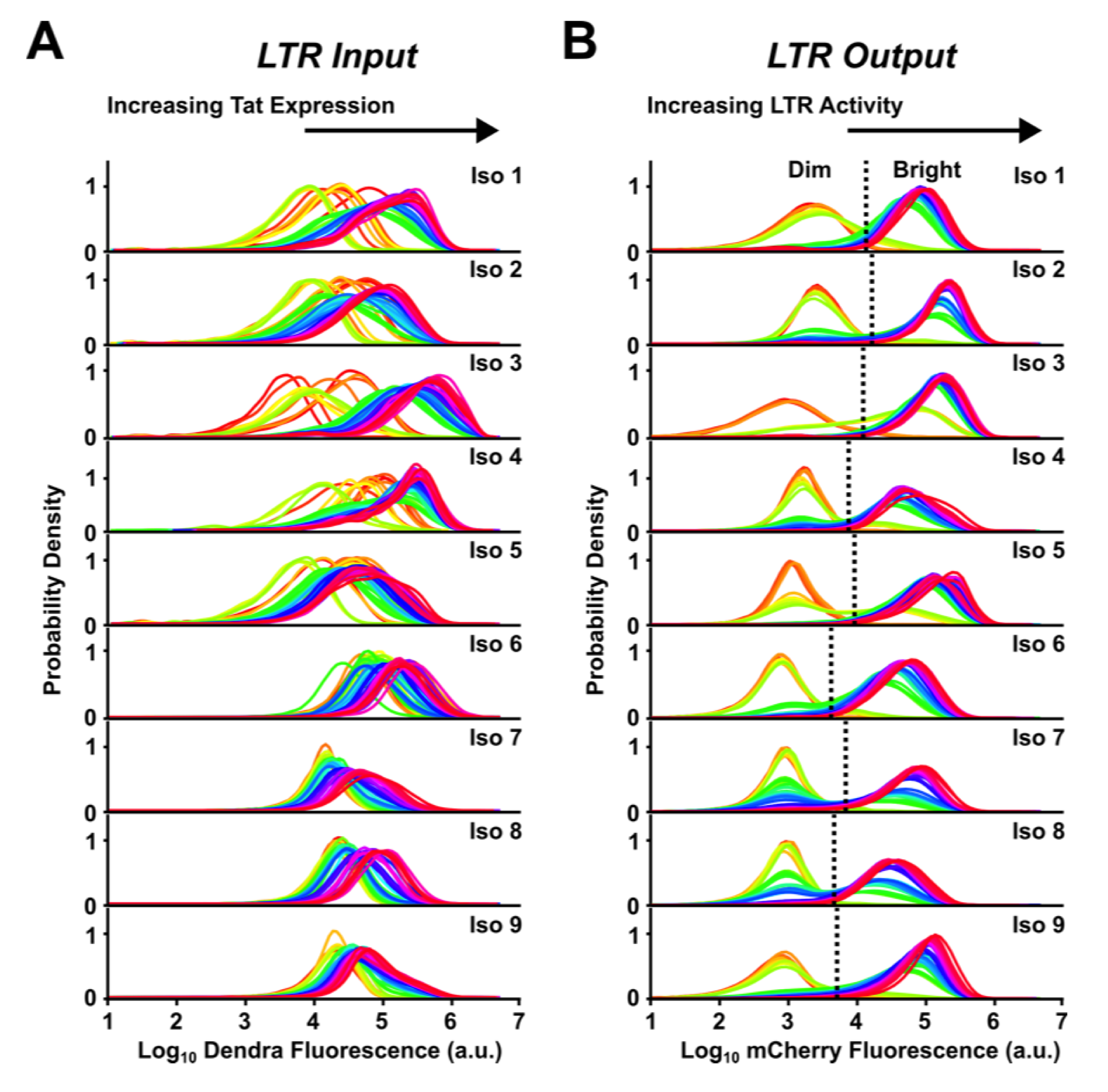

Supplement: S2 Fig — (A) Histograms of the transactivator of transcription (Tat) input to the LTR, as measured by Dendra fluorescent signal, are unimodal across all combinations of doxycycline and Shield-1. The colors of the lines indicate increasing doxycycline concentrations (red, 0 ng/mL → orange, 2.5 ng/mL → yellow, 5 ng/mL → green, 12.5 ng/mL → cyan, 25 ng/mL → blue, 50 ng/mL → pink, 250 ng/mL → magenta, 500 ng/mL), and the increasing brightness of the same color represents increasing Shield-1 concentrations (0, 10, 50, 100, 500, and 1,000 nM). (B) Histograms of LTR output as measured by mCherry fluorescent signal. The “Dim”/“Bright” threshold was set based on each population’s mCherry expression in the absence of doxycycline or Shield-1 (i.e., no Tat). The change in signal in the Bright population was used to determine the small-signal loop gain (S12 Fig) in response to Tat. The graphs were generated by ks-density clustering of the data, which can smooth features of a rough distribution, exaggerating particular features. Noticeably, some of the seemingly bimodal distributions do not pass the quantitative metrics used in Figs 2 and 3 and S3 and S7 Figs (S1–S24 Data). (TIF) [file pbio.2000841.s037.tif]

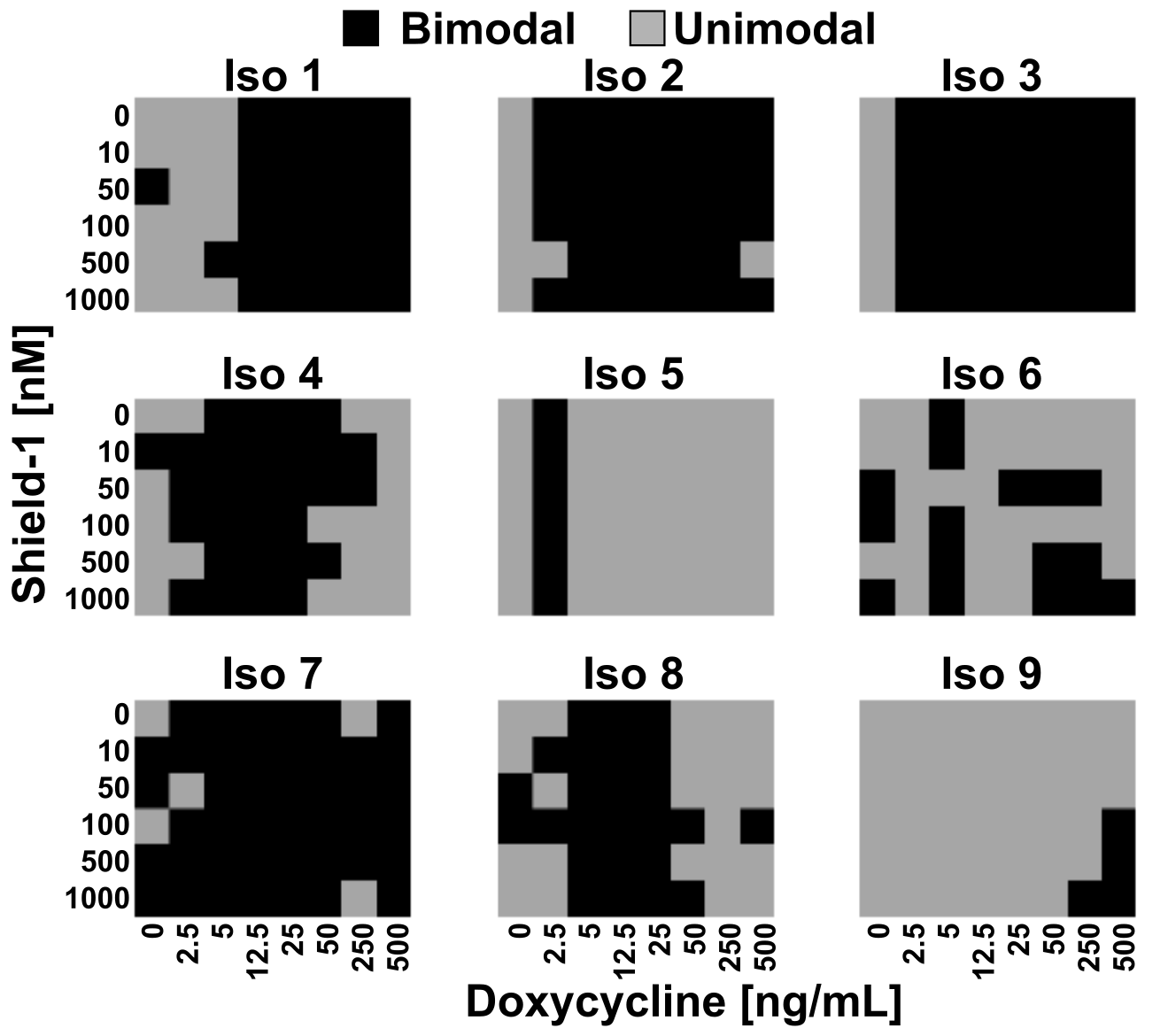

Supplement: S3 Fig — Nine isolconal populations of the open-loop circuits described in Fig 2 were exposed to 48 different doxycycline or Shield-1 concentrations. The populations were assessed for the number of modes as described in the Materials and methods section. Briefly, fluorescence intensity data were smoothed using the bkde function in the KernSmooth package in R to a binned kernel density. The number of modality peaks was calculated by taking the second-order derivative of the kernel density. Gray squares are unimodal, and black squares are bimodal (S22 and S23 Data). (TIF) [file pbio.2000841.s038.tif]

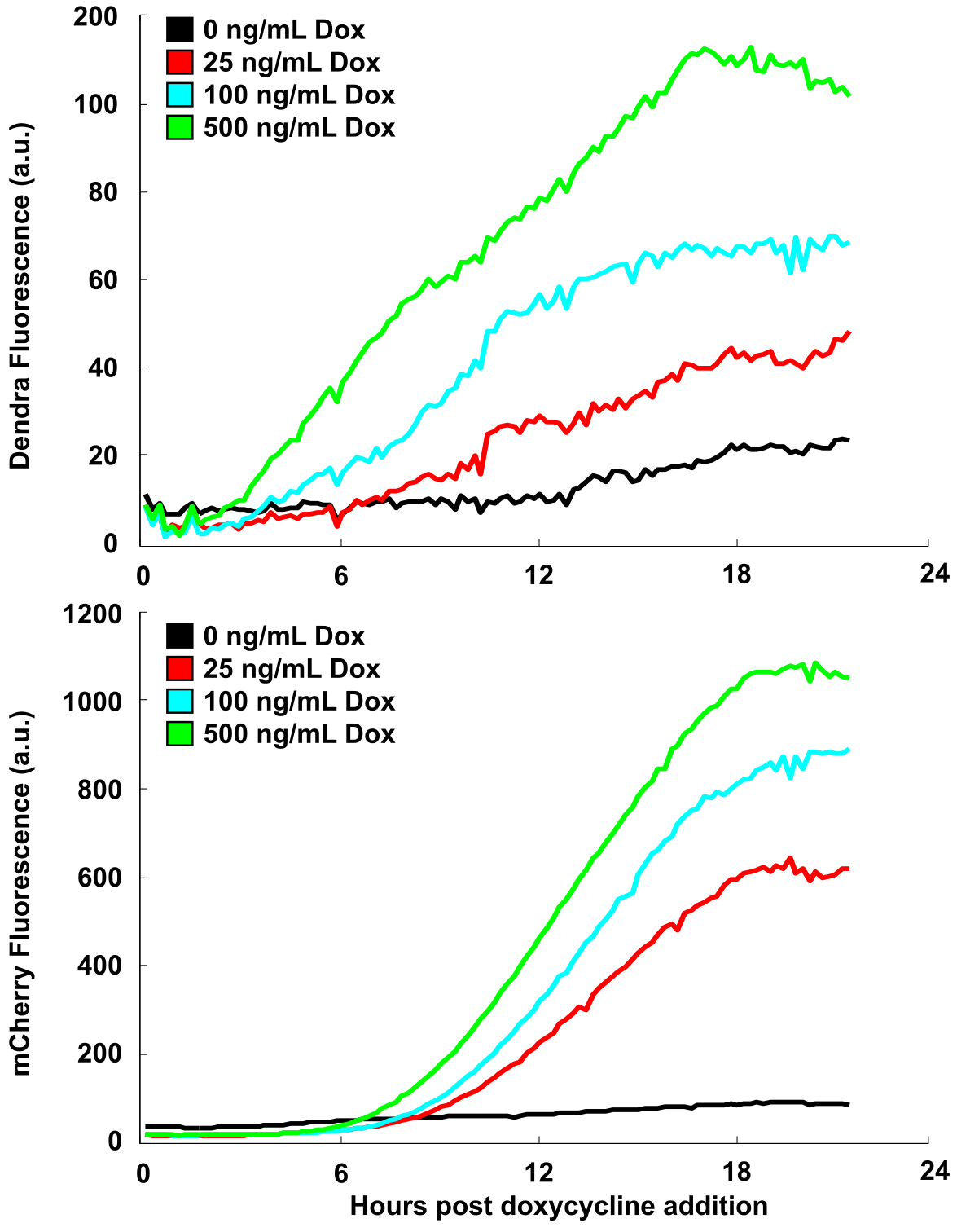

Supplement: S4 Fig — Single-cell time-lapse fluorescence microscopy of the open-loop circuit without doxycycline (black lines) or with 25 ng/mL (red lines), 100 ng/mL (cyan lines), or 500 ng/mL (green lines) of doxycycline. Both Dendra (i.e., transactivator of transcription (Tat) levels) and mCherry (i.e., LTR activity) fluorescence levels were tracked over time. Variable Tat inputs as measured by Dendra fluorescence lead to variable expression pulses from the LTR as measured by Cherry expression (S24 Data). (TIF) [file pbio.2000841.s039.tif]

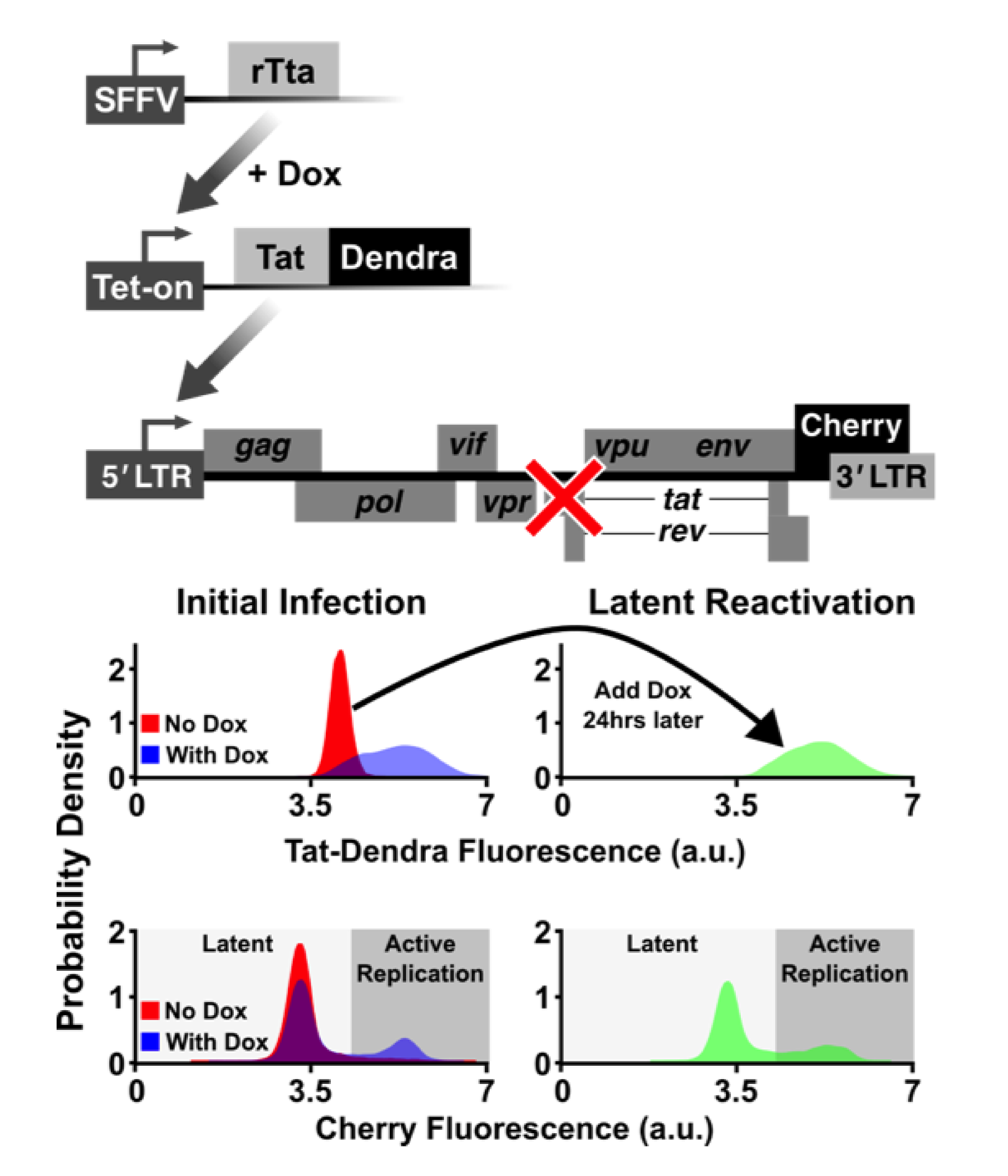

Supplement: S5 Fig — Schematic of the full-length HIV open-loop circuit (top). Doxycycline addition induces transactivator of transcription (Tat) expression, which can activate expression of the full-length HIV virus with a fluorescent mCherry reporter. Cells were initially infected in the absence (red histogram) or presence (blue histogram) of doxycycline, and a time point was taken 24 hours post infection (left side, “Initial Infection”). Doxycycline was then added to a split of the “No Dox” at the Initial Infection for 24 hours to look for HIV reaction (right side, “Latent Reactivation”). (TIF) [file pbio.2000841.s040.tif]

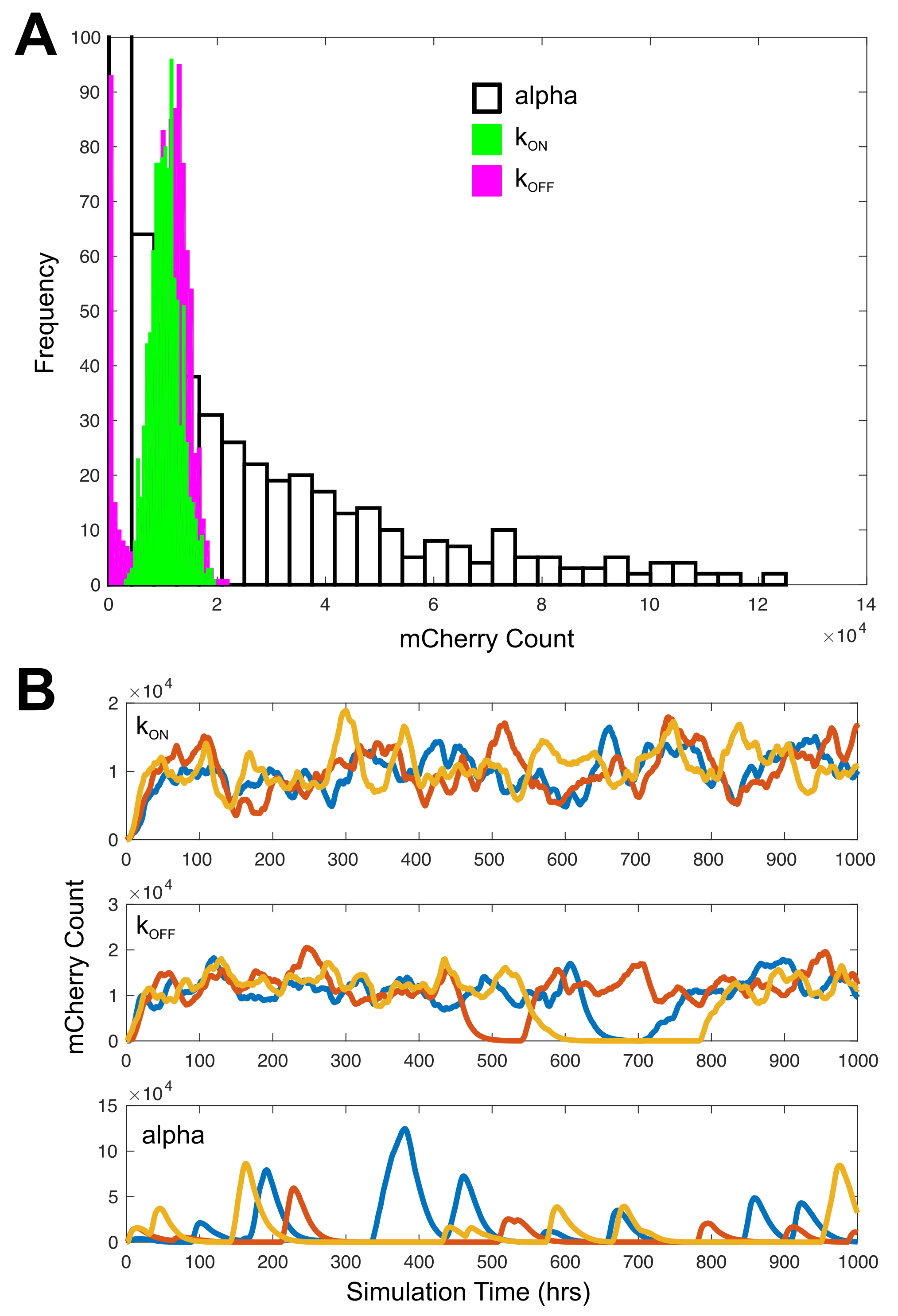

Supplement: S6 Fig — (A) Each parameter set was allowed to run for 1,000 stochastic simulations, where Tat would work through kON (green), kOFF (pink), or alpha (black lined) alone. The average protein count is equivalent for all the simulations. (B) The time course of the mCherry count over time, showing the extent of stochastic fluctuations when Tat affects kON, kOFF, or alpha. Three representative traces are shown for each (S1 Data). (TIF) [file pbio.2000841.s041.tif]

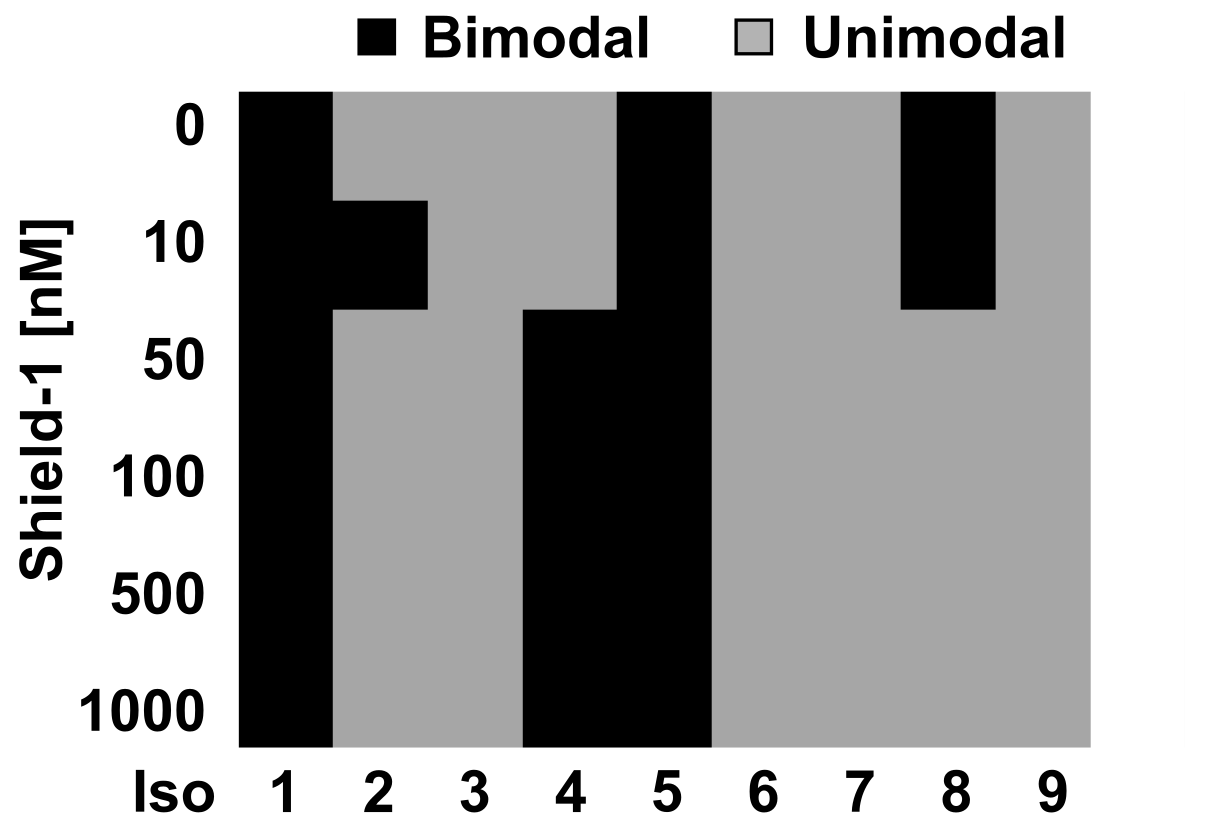

Supplement: S7 Fig — Nine isoclonal populations were exposed to various concentrations of Shield-1 as described in Fig 3. The number of modes was determined as described in the Materials and methods section. Briefly, fluorescence intensity data were smoothed using the bkde function in the KernSmooth package in R to a binned kernel density. The number of modality peaks was calculated by taking the second-order derivative of the kernel density. Gray squares are unimodal, and black squares are bimodal (S30 Data). (TIF) [file pbio.2000841.s042.tif]

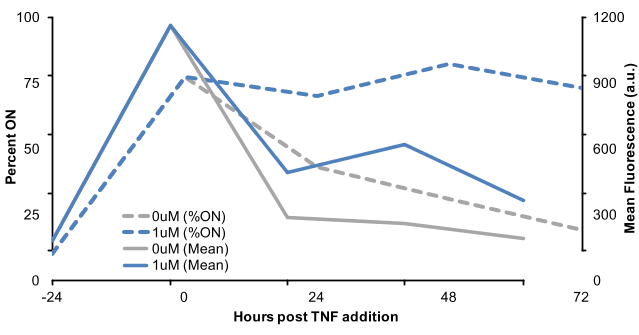

Supplement: S8 Fig — A polyclonal population of Ld2GITF (positive feedback loop expressing GFP) cells were exposed to tumor necrosis factor alpha (TNFα) for 24 hours (−24 to 0 hours), and then the cells were washed and split into 1 cultures, 1 with Shield-1 (1 uM, blue) and 1 in the absence of Shield-1 (0 uM, gray). Green fluorescent protein (GFP) measurements were taken every 24 hours, and the mean fluorescence intensity (right axis) or the percentage of cells in the ON state (left axis) was quantified. In the absence of Shield-1 after 72 hours, the cells returned to the unperturbed state in both percent ON and mean fluorescence intensity. In the presence of Shield-1, positive-feedback strength is increased, and the system remains activated for a longer duration of time. Importantly, both populations return to the state of no TNFα addition, i.e., no bistability (S31 Data). (TIF) [file pbio.2000841.s043.tif]

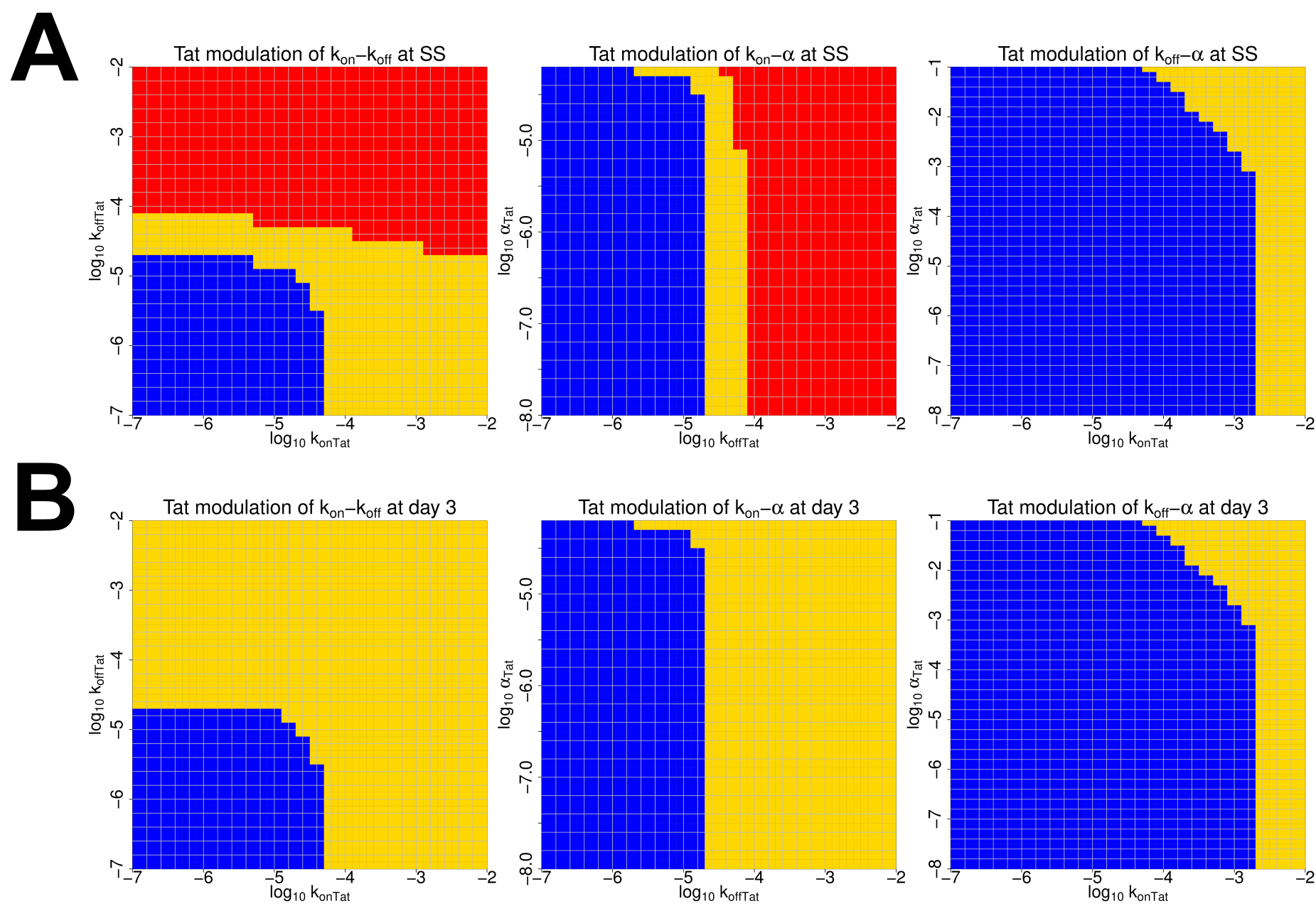

Supplement: S9 Fig — We consider 3 different phenotypes: unimodality of latency (blue areas), unimodality of activation (red areas), and bimodality (yellow areas). The phase diagrams of phenotypes for 3 different modulations based on the steady-state probability landscapes—kON-kOFF (left graphs), kON-alpha (middle graphs), and kOFF-alpha (right graphs)—are shown in part A, and the phenotype phase diagrams based on the day 3 probability landscapes are shown in part B. Details about the models and parameter sweeping can be found in the Materials and methods section. In the modulations of kON-kOFF (left graphs) and kON-alpha (middle graphs), some parameter pairs are bimodal at day 3 (yellow area in part B) but become unimodality of activation at the steady state (red area in part A). This is due to the slow evolution of the probability landscape in these parameter pairs. The phenotypes of all parameter pairs in the kOFF-alpha (right graphs) modulation at steady state are consistent with those at day 3. All simulations were started with initial toggling kinetics of kON = 0.001/min, kOFF = 0.01/min, and the rest of the parameters can be found in S1–S3 Tables. (TIF) [file pbio.2000841.s044.tif]

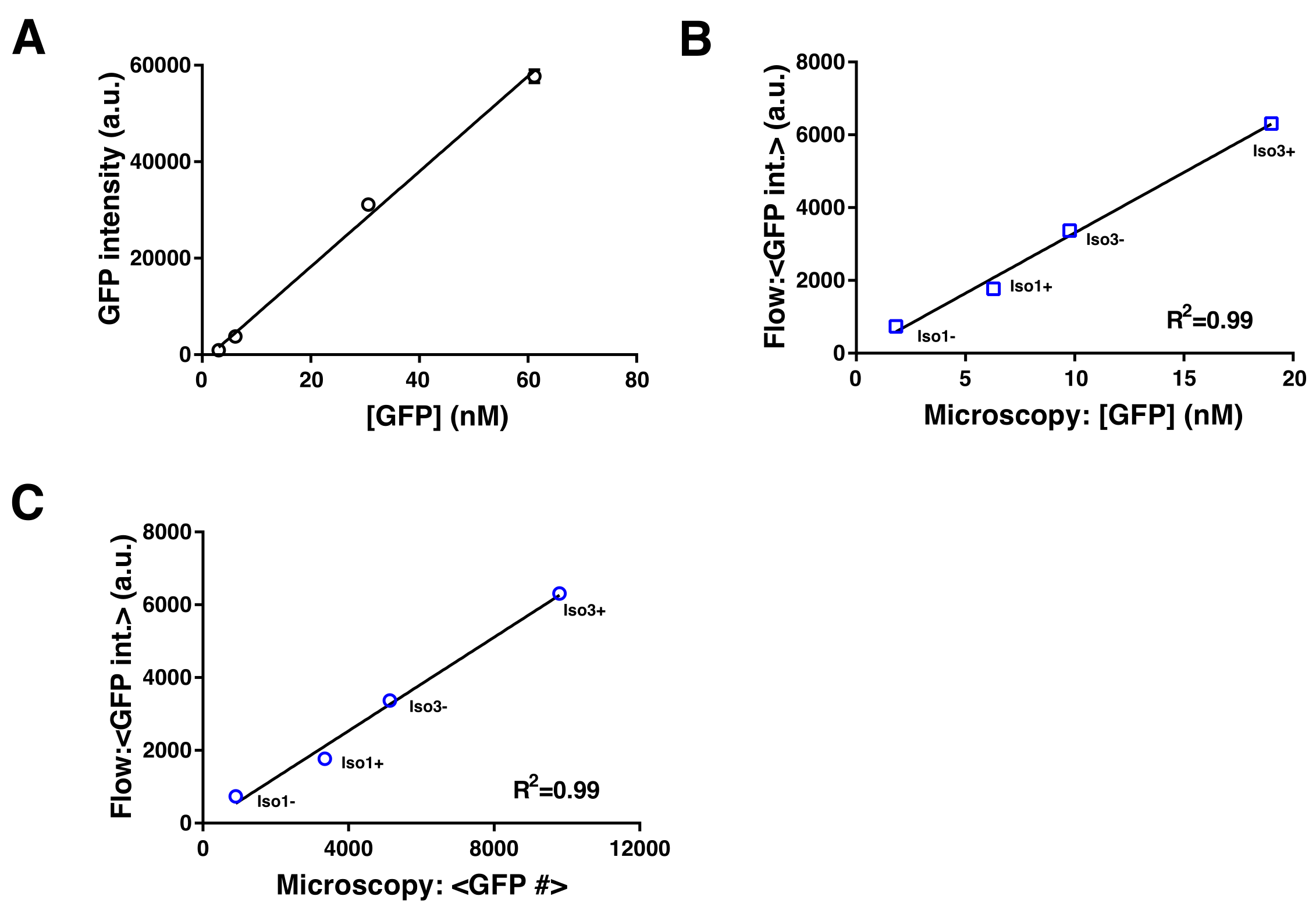

Supplement: S10 Fig — (A) Enhanced GFP (eGFP) calibration curve; dilutions of soluble recombinant eGFP protein were imaged by confocal microscopy. (B) Confocal microscopy (using the same microscope settings as in panel A) and flow cytometry showing the mean fluorescence intensity for 2 isoclonal populations (Iso 1 and Iso 3) of Jurkat Ld2GITF (positive feedback loop expressing GFP) cells—containing a single integration of the Ld2GITF (LTR-d2GFP-IRES-Tat-FKBP) construct—incubated in the presence (+) and absence (−) of Shield-1 (active or inactive feedback, respectively). The GFP levels fall well within the linear regime found in panel A. (C) Mean flow cytometry GFP intensity compared to mean cellular GFP number (calculated via approximate cellular volume) showing a linear relationship (R2 = 0.99) (S33 and S34 Data). (TIF) [file pbio.2000841.s045.tif]

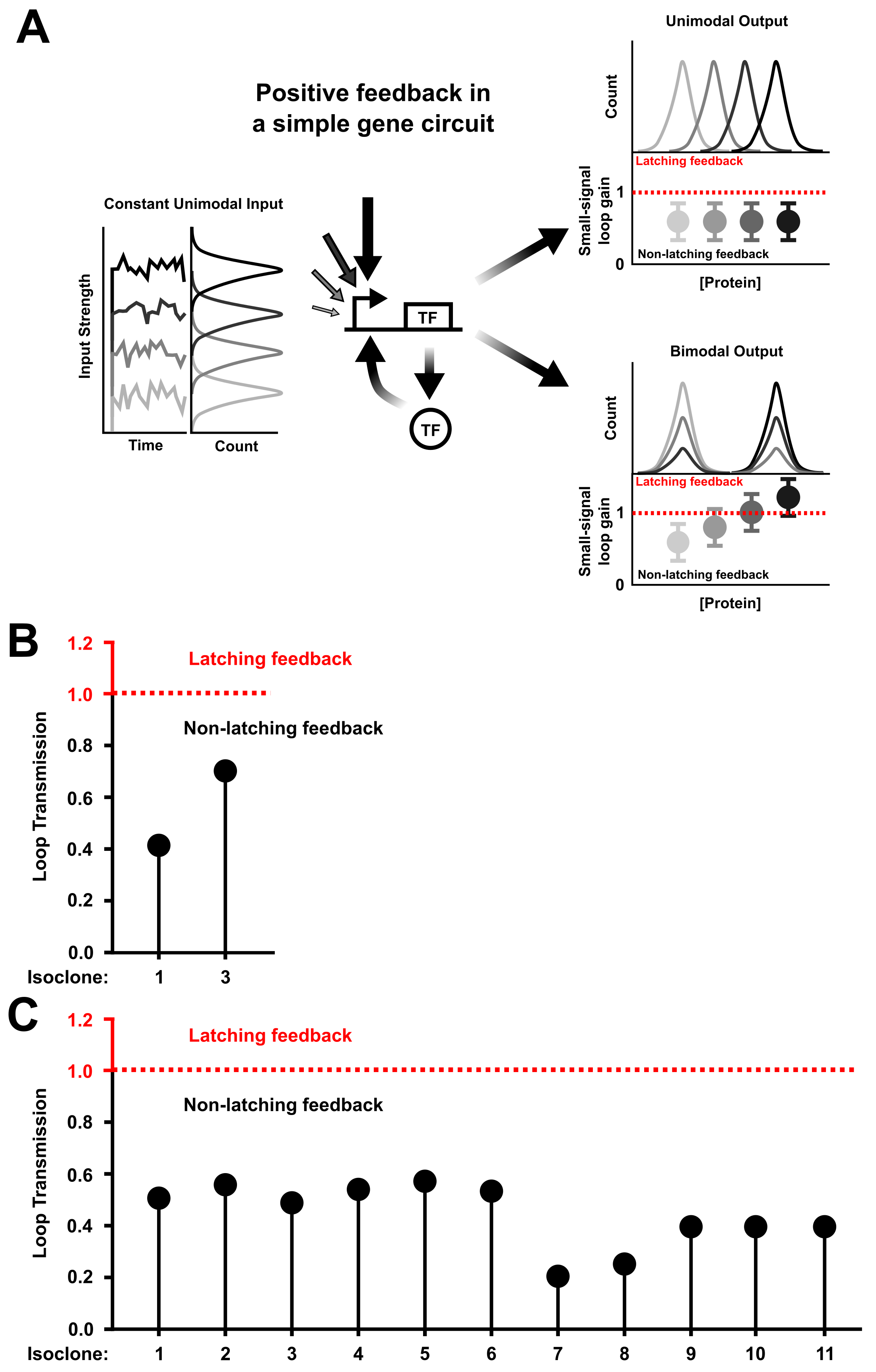

Supplement: S11 Fig — (A) A schematic showing the input-output relationship for a positive-feedback loop under the control of a constitutive promoter. Unimodal signal inputs of varying strengths reach a constitutive promoter encoding for a transcription factor (TF), which initiates positive feedback. The level of amplification due to positive feedback is quantified by the small-signal loop gain. For loop gains < 1 across all protein concentrations, the system displays nonlatching feedback and the results in a unimodal output over the abundance regime. However, if small-signal loop gain increases with protein abundance to approximately 1, small input fluctuations are drastically amplified and can generate a bimodal distribution in the output (bottom right). The error bars around the circles in A (right-hand graphs) represent, for a population of cells that receive the same inputs, the fluctuations that would lead some cells to display higher or lower small-signal loop gains. (B) Quantification of the small-signal loop gain of the closed-loop circuit for the 2 isoclonal Ld2GITF populations used in S10 Fig—to verify that Tat feedback is nonlatching in the linear fluorescence-to-protein regime. (C) Quantification of the small-signal loop gain for the closed-loop circuits of the 9 isoclonal LChITF populations used in Fig 3 showing that Tat feedback is nonlatching (S1–S25 Data). (TIF) [file pbio.2000841.s046.tif]

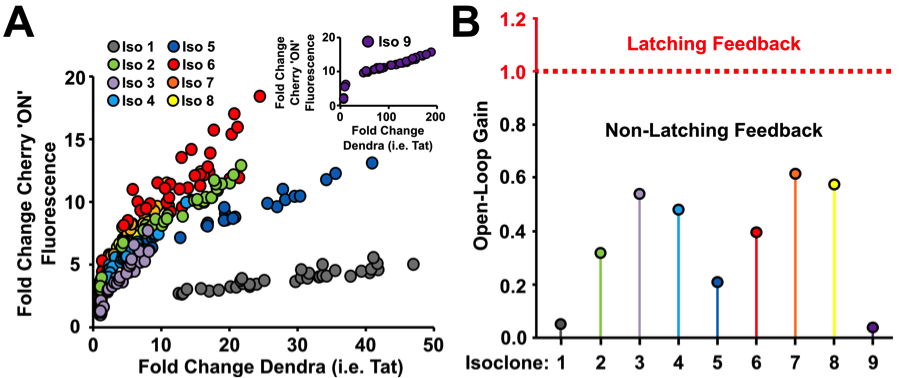

Supplement: S12 Fig — (A) Plot of the fold change in transactivator of transcription (Tat)-Dendra abundance versus the fold change in mCherry ON population expression for 9 isoclonal populations. (B) Quantification of the small-signal open-loop gain of the 9 isoclonal populations. These values are representative of the expected small-signal loop gain for an intact circuit with feedback. Importantly, all 9 isoclonal populations indicate that Tat feedback is nonlatching (S32 Data). (TIF) [file pbio.2000841.s047.tif]

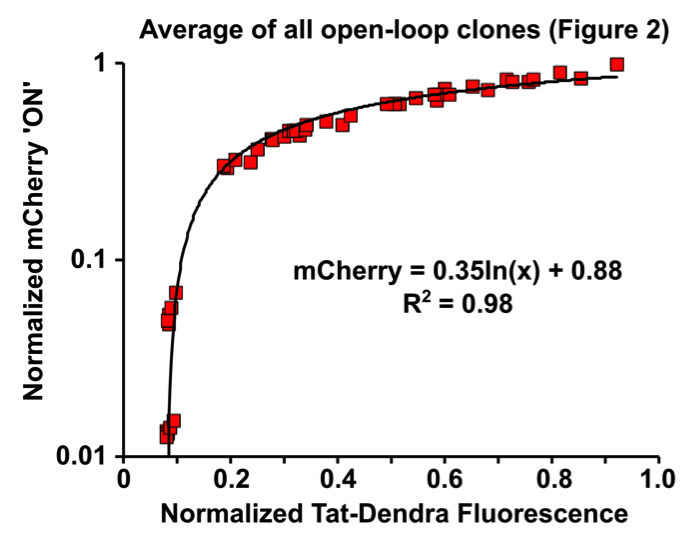

Supplement: S13 Fig — Plot of normalized LTR-mCherry output to normalized Tat-Dendra fluorescence for the 11 clonal populations (Fig 2). The data are best fit with a logarithmic function but can also be represented with 2 linear fits (R2 = 0.98): 1 fit for the sensitive region (between 0 and 0.2, Normalized Tat-Dendra Fluorescence) and 1 fit for the insensitive region (between 0.2 and 1, Normalized Tat-Dendra Fluorescence) (S1–S22 Data). (TIF) [file pbio.2000841.s048.tif]

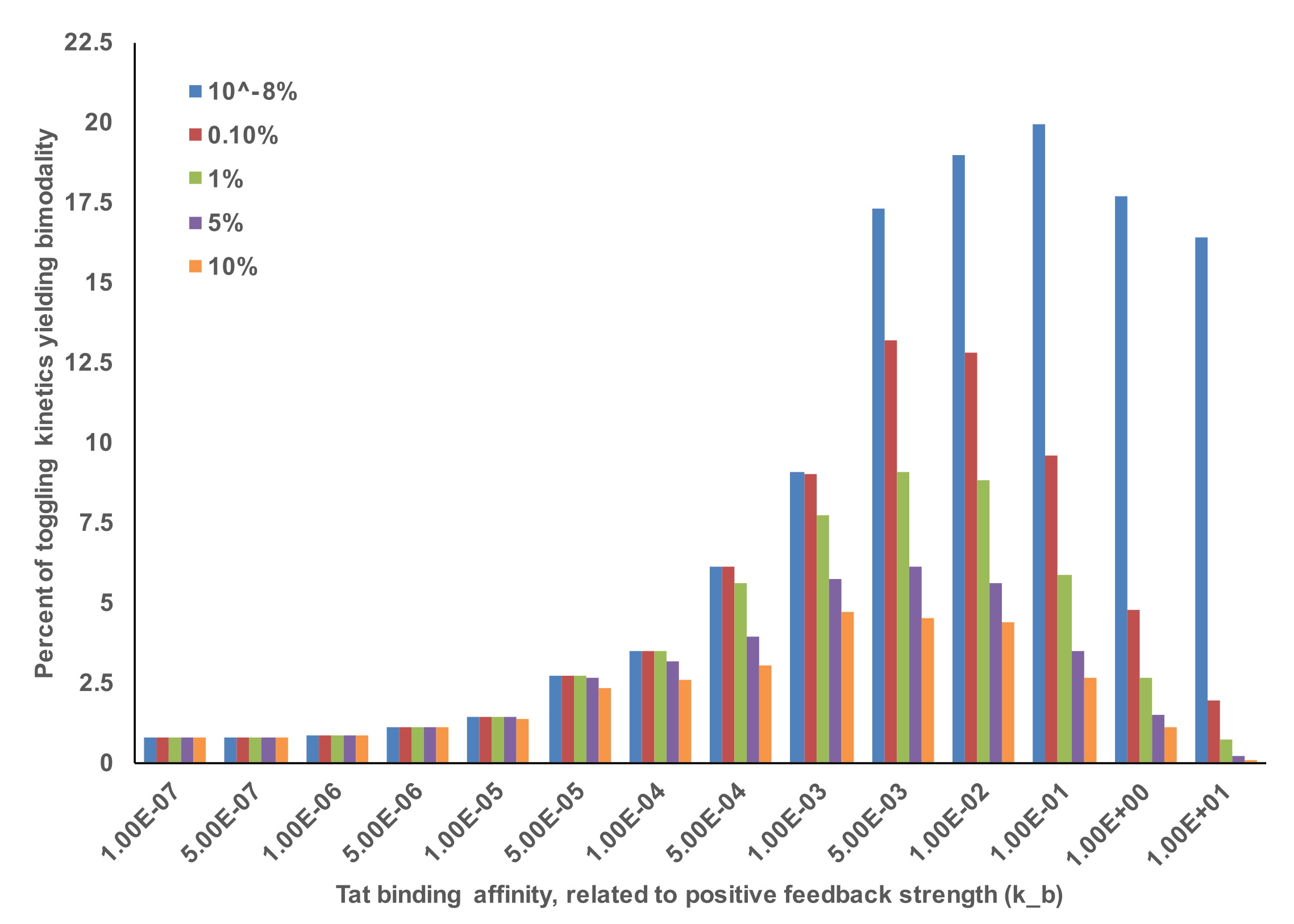

Supplement: S14 Fig — To simulate various feedback strengths, the binding affinity, kb, was tuned from 5 x 10^−7^ to 10, on a parameter scan across kON and kOFF values ranging from 0.001 to 10/minute. Next, of those parameter scans, a bimodality test was performed (Materials and methods). The percentage of parameters that yielded bimodality was then quantified. Various thresholds were set to determine whether a population was bimodal by requiring that each mode had to have 10−8%, 0.1%, 1%, 5%, or 10% of the total population (S35 Data). (TIF) [file pbio.2000841.s049.tif]
