# Supplementary material for: Nonlatching positive feedback enables robust bimodality by decoupling expression noise from the mean
Source: PLoS Biol. 2017 Oct 18;15(10):e2000841. doi: 10.1371/journal.pbio.2000841 (PMC5646755; doi:10.1371/journal.pbio.2000841)
Supplement: S1 Table — For the open-loop circuit with no positive feedback, the ** reaction is present, but the * reaction is not. The variable input in the ** parameter represents the different transactivator of transcription (Tat) inputs, which experimentally are varied by adding different amounts of doxycycline to the culture (Fig 2). The * reaction closes the loop and is used to model the circuit that has positive feedback. The ** reaction is not used for the models with positive feedback. The fifth reaction defines Tat’s modulation of promoter toggling. For Tat affecting kOFF, Tat binds to the LTRON state and creates a third state, LTRTatON. From the LTRTatON state, the LTR must move through LTRON first before switching back OFF. (PDF) [file pbio.2000841.s050.pdf]

Reactions	***Description***	***Rate and Value***
$LTR_{OFF} \leftrightarrow LTR_{ON}$	Promoter toggling from active to inactive state (basal transcription rate)	k_{on} = variable; k_{off} = variable
$LTR_{ON} \rightarrow mRNA + LTR_{ON}$	Transcription of mRNA encoding Tat	α= 1
mRNA → mRNA + mCherry	Translation	k_p = 10
*mRNA → mRNA + Tat	Translation	k_p = 10
$Tat + LTR_{ON} \leftrightarrow Tat\text{-}LTR_{ON}$	Tat switching between the Tat-dependent and Tat-independent 'ON' state	kTatbind = variable<br>kTatOFF = variable
$Tat\text{-}LTR_{ON} \rightarrow Tat\text{-}LTR_{ON}$ + mRNA	Transcription from Tat-activated state	α= 1
mRNA → 0	mRNA decay	d_m = .043
Tat → 0	Tat decay	d_m = .0024
mCherry → 0	mCherry decay	d_p = 0.008
**Tat_{init}	Steady-state Tat input; for open-loop simulations	Variable (0-10,000)
