# Supplementary material for: Nonlatching positive feedback enables robust bimodality by decoupling expression noise from the mean
Source: PLoS Biol. 2017 Oct 18;15(10):e2000841. doi: 10.1371/journal.pbio.2000841 (PMC5646755; doi:10.1371/journal.pbio.2000841)
Supplement: S2 Table — See the S1 Table description for further information. The fifth reaction represents Tat’s ability to modulate burst frequency through kON. Tat binds to the LTROFF state and flips the promoter to the LTRON state. (PDF) [file pbio.2000841.s051.pdf]

Reactions	***Description***	***Rate and Value***
$LTR_{OFF} \leftrightarrow LTR_{ON}$	Promoter toggling from active to inactive state (basal transcription rate)	k_{on} = variable; k_{off} = variable
$LTR_{ON} \rightarrow mRNA + LTR_{ON}$	Transcription of mRNA encoding Tat	α= 1
mRNA → mRNA + mCherry	Translation	k_p = 10
*mRNA → mRNA + Tat	Translation	k_p = 10
$Tat + LTR_{OFF} \rightarrow Tat + LTR_{ON}$	Tat switching the LTR to 'ON' state	k_{TatON} = variable
mRNA → 0	mRNA decay	d_m = .043
Tat → 0	Tat decay	d_m = .0024
mCherry → 0	mCherry decay	d_p = 0.008
**Tat_{init}	Steady-state Tat input; for open-loop simulations	Variable (0-10,000)
