# Supplementary material for: Nonlatching positive feedback enables robust bimodality by decoupling expression noise from the mean
Source: PLoS Biol. 2017 Oct 18;15(10):e2000841. doi: 10.1371/journal.pbio.2000841 (PMC5646755; doi:10.1371/journal.pbio.2000841)
Supplement: S3 Table — See the S1 Table description for further information. The fifth reaction defines Tat’s modulation of burst size by modulating alpha. Tat binds to the LTRON state and promotes transcription, thereby affecting transcriptional efficiency when the promoter is already in an active state. (PDF) [file pbio.2000841.s052.pdf]

Reactions	***Description***	***Rate and Value***
$LTR_{OFF} \leftrightarrow LTR_{ON}$	Promoter toggling from active to inactive state (basal transcription rate)	k_{on} = variable; k_{off} = variable
$LTR_{ON} \rightarrow mRNA + LTR_{ON}$	Transcription of mRNA encoding Tat	$\alpha = 1$
mRNA $\rightarrow$ mRNA + mCherry	Translation	$k_p = 10$
*mRNA $\rightarrow$ mRNA + Tat	Translation	$k_p = 10$
Tat + $LTR_{ON} \rightarrow LTR_{ON}$ + mRNA + Tat	Tat induction of transcription	α_{Tat} = variable
mRNA $\rightarrow$ 0	mRNA decay	$d_m = .043$
Tat $\rightarrow$ 0	Tat decay	$d_m = .0024$
mCherry $\rightarrow$ 0	mCherry decay	$d_p = 0.008$
**Tat_{init}	Steady-state Tat input; for open-loop simulations	Variable (0-10,000)
