# Supplementary material for: Nonlatching positive feedback enables robust bimodality by decoupling expression noise from the mean
Source: PLoS Biol. 2017 Oct 18;15(10):e2000841. doi: 10.1371/journal.pbio.2000841 (PMC5646755; doi:10.1371/journal.pbio.2000841)
Supplement: S1 Text — The supplementary text provides the derivation and assumptions behind Eq 1 and Eq 2 and Fig 5 of the main text. (DOCX) [file pbio.2000841.s053.docx]

**Section 1.** The effect of feedback on bimodality robustness

To model the probability of crashing into the OFF state, we assume that the ON state Tat population (x) has a normal distribution such that

$$Prob\left( x:\mu, \sigma^{2} \right)=\frac{1}{\sqrt{2\pi\sigma^{2}}}e^{-\frac{\left( x-\mu\right)^{2}}{2\sigma^{2}}}$$

where µ and σ are the average and standard deviation of the Tat population in the ON state. The transition into the OFF state is triggered by extinction of Tat, and the probability of this crashing may be calculated as

${Prob}_{crash}=\int_{-\infty}^{0} \frac{1}{\sqrt{2\pi\sigma^{2}}}e^{-\frac{\left( x-\mu\right)^{2}}{2\sigma^{2}}}dx$.

In general in the absence of feedback, σ^2^(Tat variance) varies linearly with µ, and the probability of crashing rapidly decreases with an increasing average Tat population.

In contrast, positive feedback alters the relationship between the Tat variance and mean such that $\sigma^{2}\propto\mu^{N}$, where N>1. For example, for linear non-latching positive feedback (Simpson et al., 2003)

$$\mu=\frac{\mu_{NFB}}{1-T}$$

where $\mu_{NFB}$ is the average Tat population that would have occurred in the absence of feedback, and T is a measure of the strength of the positive feedback known as the loop transmission. Furthermore (Simpson et al., 2003)

$$\sigma^{2}=\frac{{\sigma^{2}}_{NFB}}{\left( 1-T \right)^{2}}$$

where ${\sigma^{2}}_{NFB}$ is the Tat variance that would have occurred in the absence of feedback. So for this idealized case of positive feedback, $\sigma^{2}\propto\mu^{2}$, and

${Prob}_{crash}=\int_{-\infty}^{0} \frac{\left( 1-T \right)}{\sqrt{2\pi{\sigma^{2}}_{NFB}}}e^{-\frac{\left( x-\frac{\mu_{NFB}}{1-T} \right)^{2}}{2\frac{{\sigma^{2}}_{NFB}}{\left( 1-T \right)^{2}}}}dx$.

With the substitution of variable

$$v=\left( 1-T \right)x$$

$${Prob}_{crash}=\int_{-\infty}^{0} \frac{1}{\sqrt{2\pi{\sigma^{2}}_{NFB}}}e^{-\frac{\left( v-\mu_{NFB} \right)^{2}}{2{\sigma^{2}}_{NFB}}}dv$$

which gives the same probability of crashing into the OFF state regardless of the feedback mediated change in the average Tat population.

For non-idealized positive feedback, $\sigma^{2}\propto\mu^{N}$ (1<N<2) and bimodal behavior becomes more robust as N approaches 2. For the LTR-Tat positive feedback circuit N~1.5, and bimodality is significantly more robust than that found for non-feedback architectures (Figure 5 in the main text).

**REFERENCES**

Simpson, M.L., Cox, C.D., Sayler, G.S., 2003. Frequency domain analysis of noise in autoregulated gene circuits. Proc Natl Acad Sci USA 100, 4551–4556.
